# miR-34a is a tumor suppressor in zebrafish and its expression levels impact metabolism, hematopoiesis and DNA damage

**DOI:** 10.1371/journal.pgen.1011290

**Published:** 2024-05-28

**Authors:** Sergey V. Prykhozhij, Kevin Ban, Zane L. Brown, Kim Kobar, Gabriel Wajnberg, Charlotte Fuller, Simi Chacko, Jacynthe Lacroix, Nicolas Crapoulet, Craig Midgen, Adam Shlien, David Malkin, Jason N. Berman

**Affiliations:** 1 Children’s Hospital of Eastern Ontario (CHEO) Research Institute and University of Ottawa, Ottawa, Ontario, Canada; 2 Dalhousie University Medical School, Halifax, Nova Scotia, Canada; 3 Atlantic Cancer Research Institute, Pavillon Hôtel-Dieu, 35 Providence Street, Moncton, NB, Canada; 4 HHS McMaster University Medical Centre, Division of Medical Microbiology, Hamilton, Ontario, Canada; 5 Atlantic Cancer Research Institute, Pavillon Hôtel-Dieu, Moncton, New Brunswick, Canada; 6 Department of Pathology, Dalhousie University, Halifax, Nova Scotia, Canada; 7 IWK Health Centre, Halifax, Nova Scotia, Canada; 8 Genetics and Genome Biology Program, The Hospital for Sick Children, PGCRL, Toronto, Ontario, Canada; 9 Departments of Pediatrics and Medical Biophysics, University of Toronto, Toronto, Ontario, Canada; Fred Hutchinson Cancer Research Center, UNITED STATES

## Abstract

Li-Fraumeni syndrome is caused by inherited *TP53* tumor suppressor gene mutations. MicroRNA *miR-34a* is a p53 target and modifier gene. Interestingly, *miR-34* triple-null mice exhibit normal p53 responses and no overt cancer development, but the lack of miR-34 promotes tumorigenesis in cancer-susceptible backgrounds. *miR-34* genes are highly conserved and syntenic between zebrafish and humans. Zebrafish *miR-34a* and *miR-34b*/*c* have similar expression timing in development, but *miR-34a* is more abundant. DNA damage by camptothecin led to p53-dependent induction of *miR-34* genes, while *miR-34a* mutants were adult-viable and had normal DNA damage-induced apoptosis. Nevertheless, *miR-34a*-/- compound mutants with a gain-of-function *tp53*^*R217H/ R217H*^ or *tp53-/-* mutants were more cancer-prone than *tp53* mutants alone, confirming the tumor-suppressive function of *miR-34a*. Through transcriptomic comparisons at 28 hours post-fertilization (hpf), we characterized DNA damage-induced transcription, and at 8, 28 and 72 hpf we determined potential miR-34a-regulated genes. At 72 hpf, loss of *miR-*34a enhanced erythrocyte levels and up-regulated *myb*-positive hematopoietic stem cells. Overexpression of miR-34a suppressed its reporter mRNA, but not p53 target induction, and sensitized injected embryos to camptothecin but not to γ-irradiation.

## Author summary

Li-Fraumeni syndrome, rooted in inherited mutations of the *TP53* tumor suppressor gene, presents a complex interplay with *microRNA34a* (*miR-34a*), a known p53 target and modifier gene. Notably, *miR-34* triple-null mice, despite exhibiting typical p53 responses and no evident cancer development, unveil a crucial role for *miR-34* genes in suppressing tumorigenesis, particularly in predisposed conditions. The evolutionary conservation of *miR-34* genes across zebrafish and humans underscores their significance. Studying zebrafish embryos reveals intriguing insights into miR-34a dynamics during development and its response to DNA damage, illuminating its involvement in cancer susceptibility. Additionally, experiments involving *miR-34a* mutants further confirm its tumor-suppressive function, especially when coupled with *tp53* mutations. Transcriptomic analysis at different developmental stages uncover potential genes regulated by miR-34a, shedding light on its broader regulatory role. These findings enhance our understanding of the intricate mechanisms underlying cancer susceptibility and highlight miR-34a as a potential therapeutic target for Li-Fraumeni syndrome and related malignancies.

## Introduction

The p53 tumor suppressor is essential for cellular homeostasis by responding to DNA damage and other potentially oncogenic stimuli [1]. Under normal conditions, p53 protein levels are tightly controlled; however, stressors (e.g. DNA damage, oncogene activation) stabilize and modify p53 to activate it and increase its levels [1]. Upon activation, p53 translocates to the nucleus and activates its target genes [2], which regulate key genes involved in cell cycle arrest, senescence, apoptosis, DNA repair, autophagy, transcriptional regulation, metabolism, and the regulation of p53 [1,3]. *TP53* mutations cause cancer predisposition, are found in most sporadic cancer types at about 30% mean frequency, and germline *TP53* mutations are the primary cause of the Li-Fraumeni cancer predisposition syndrome (LFS) [4].

microRNA (miRNA) genes are transcribed by RNA polymerase II, processed by Drosha and Dicer enzyme complexes, and then mature 21–25 nucleotide miRNAs associate with the RNA-induced silencing complex to target mRNA by base complementarity. This leads to mRNA translational repression by blocking ribosome recruitment or induces mRNA cleavage [5]. p53 transcriptionally up-regulates many miRNA genes: the miR-34 family of miRNAs and more than 20 other miRNA genes; while a few miRNA genes, such as the miR-17/92 cluster are down-regulated by p53 [6]. Moreover, p53 itself is regulated by more than 20 microRNAs such as miR-125b which negatively controls p53 levels [7].

The *miR-34* gene family in most vertebrates consists of *miR-34a* and *miR-34b/c* genes. The *miR-34a* gene is expressed nearly ubiquitously and in most tissues at a much higher level than *miR-34b/c*, which is expressed predominantly in the mammalian brain, lungs, testes and fallopian tubes [8]. *MIR34A* was the first microRNA gene identified as a p53 target, which is consistent with evidence that miR-34a overexpression stimulates apoptosis and cell cycle arrest [9–12]. Later studies revealed that multiple types of cancer exhibit hypermethylation and down-regulation of *miR-34a* or all *miR-34* genes, as well as a worse prognosis for tumors with low miR-34 expression levels, suggesting a role of the miR-34 family as tumor suppressors [13,14]. miR-34a is also a known p53 pathway modifier in LFS, where *TP53* missense mutations were strongly associated with *MIR34A* promoter hypermethylation in tumors compared to the surrounding healthy tissue; this hypermethylation was associated with a poorer prognosis in choroid plexus carcinoma patients [15]. Additional strong evidence for the role of miR-34a in tumor suppression came from the study by Öner et al. [16] showing a cooperation between tissue-specific deletion of Tp53 and MiR-34a in a chemically-induced mouse model of colorectal cancer. These authors also found in large patient cohorts a significant association of poorer survival of patients with primary colorectal cancer that harbor mutant *TP53* and show *MIR34A* silencing vs those who are *TP53* mutant alone. An experimental study of Kras^G12D^-mediated oncogenesis in the *tp53*^*+/-*^ mice showed that loss of Mir34a dramatically increased tumor size and frequency [17]. The mouse Adenomatous Polyposis Coli (APC) mutant intestinal cancer model (Apc^Min/+^) revealed both *Mir34a* and *Mir34b/Mir34c* genes were required for tumor suppression since loss of all three Mir34s increased tumor size and aggressiveness [18].

miR-34a performs its functions by inhibiting translation and stability of its target mRNAs, which encode proteins involved in cell cycle, apoptosis and senescence, epithelial-to-mesenchymal transition (EMT), metastasis, stemness and metabolism [19]. The inhibitory effects of miR-34a on cell-cycle progression are mediated via down-regulation of cyclin D1 and E2, cyclin-dependent kinases CDK4 and CDK6, as well as of c-Myc, N-Myc and E2F Transcription Factor 3 (E2F3). Apoptosis is generally induced by overexpression of miR-34a due to repression of multiple anti-apoptotic genes (e.g. *BCL2, BIRC5, XIAP1, ALDH2, ATF1, YY1*) [19]. miR-34a also inhibits negative regulators of p53, such as HDM4 [17], MDM4 [20] and SIRT1 [21], thus augmenting the p53 response. Further, inactivation of miR-34a was shown to attenuate the p53 response, and overexpression of miR-34a appeared to increase apoptosis [10,12]. Navarro and Lieberman challenged the simple positive miR-34a-p53 feedback model by showing that about a quarter of p53 target genes are direct targets of miR-34a, that both miR-34a and p53 have independent functions [22], and that a lack of miR-34a in human cells did not affect the p53 response. Similarly, mice lacking all three *Mir34* genes had apparently normal p53 function [23], demonstrating that miR-34s are not essential but rather play a fine-tuning role in the p53 pathway. Thus, these studies show that there is likely context-dependence and more detailed *in vivo* animal model work is required to understand miR-34a-p53 interactions [19].

The zebrafish is highly suitable for studies of *tp53*, other tumor suppressors and cancer modeling [24]. However, little is known about the functions of miR-34 genes in this species apart from observations of miR-34a expression in specific brain regions [25]. A later study showed the maternal contribution of miR-34a, some gene expression changes and hindbrain abnormalities after its knock-down, but these observations were not linked [26]. By contrast, knock-down of miR-34b affected multi-ciliated kidney cells leading to disruption of renal development; mechanistically miR-34b acts upstream of the *myb* gene essential for kidney development [27]. Complete knock-out of *miR-34a* in zebrafish also elevates sperm motility and fertilization rate [28].

In this study, we characterized zebrafish *miR-34a* and its functional interactions with *tp53*, as well as its independent effects by performing transcriptomic, mutant and overexpression studies. We generated and employed *miR-34a* mutant zebrafish to determine the effects of its loss on gene expression under normal conditions at three stages of development, which revealed a spectrum of transcriptomic effects. We demonstrated that expression of *miR-34* genes is partially dependent on p53 under control conditions, and their induction by DNA damage is fully p53-dependent. Moreover, we found that miR-34a has small but significant transcriptomic effects in 28 hour post-fertilization (hpf) zebrafish embryos under both control and DNA damage conditions. The most direct evidence of miR-34a tumor-suppressive functions was provided by the earlier tumor onset in compound *miR-34a* and *tp53* mutants compared to the corresponding single *tp53* mutants. Transient overexpression of miR-34a induced a greater sensitivity in zebrafish embryos to camptothecin (CPT) treatment but not to γ-irradiation. CPT is a DNA damage-inducing topoisomerase I inhibitor [29], which activates p53 in zebrafish [30]. However, artificial miR-34a overexpression does not affect the activity of p53 and likely functions independently.

## Materials and methods

### Ethics statement

Work on zebrafish presented in this article was approved by the University of Ottawa Animal Care Committee (approval number: CHEOe-3168).

### Zebrafish husbandry and maintenance

Zebrafish experiments and husbandry followed standard protocols [31]. Zebrafish embryos were maintained at 28.5°C during development and as adults. Embryos were grown in 1x E3 medium. The wild-type, *miR-34a-/-* and *tp53*^*R217H/R217H*^ lines were generated in the *casper* background [32], whereas the *tp53-/-* line is in the syngeneic CG1 line background [33]. Both of these backgrounds are originally derived from zebrafish of the AB strain. The compound *miR-34a-/-; tp53-/-* line is in the mixed genetic background, whereas the *miR-34a-/-; tp53*^*R217H/R217H*^ line is in the *casper* background. The *tp53*^*R217H/R217H*^ line has been previously generated by the current authors [34] and its detailed biological description is in preparation.

### Bioinformatic tools

Alignment of miRNA sequences was performed using Clustal Omega [35] tool (https://www.ebi.ac.uk/jdispatcher/msa/clustalo) with the default settings for RNA sequences. Gene Ontology and KEGG pathways enrichment analyses were performed using DAVID [36] (https://david.ncifcrf.gov/summary.jsp) with groups of differentially-expressed genes as a query and the full list of detected genes by RNA-seq in each dataset as a background list. Gene Ontology terms were visualized in their semantic space using REVIGO tool (http://revigo.irb.hr/) [37]. We performed p53 transcription factor motif searches with the MAST tool (https://meme-suite.org/meme/tools/mast) [38] and RSAT matrix-scan tool (http://rsat.sb-roscoff.fr/matrix-scan_form.cgi) [39] using the p53 motif matrix retrieved from JASPAR (https://jaspar.genereg.net/matrix/MA0106.3/) and sequences around zebrafish *miR-34* genes retrieved from Ensembl (http://useast.ensembl.org/index.html).

### Drug treatments

Camptothecin (MilliporeSigma, C9911) (CPT) treatments were performed by adding either DMSO (control groups) or the 2 mM stock CPT solution in DMSO to fish water to achieve the indicated concentration and incubating embryos for an indicated length of time. The treated and control embryos were anesthetized and collected for RNA extraction or fixed with 4% PFA for antibody staining or *in situ* hybridization.

### TUNEL (TdT-mediated dUTP nick end-labeling) and Acridine Orange apoptosis staining

Embryos treated with DMSO or 100 nM CPT were fixed overnight in 4% PFA and then incubated in methanol for at least 2 hours at -20°C. TUNEL staining was performed using the In Situ Cell Death Detection Kit, TMR red (Roche, 12156792910) according to a previously published protocol [40]. For Acridine Orange staining, live dechorionated embryos were incubated with 2 μg/ml of Acridine Orange (AO) (MilliporeSigma, 235474) in E3 embryo medium for 30 minutes. The traces of AO were removed by 5 changes of the embryo medium. Embryos were then imaged using ZEISS Axio Zoom.V16 microscope.

### RNA extraction, cDNA synthesis and PCR

Each total RNA sample was extracted from 30–50 zebrafish embryos, single embryos or adult tissue samples by homogenizing them in 500μL Trizol reagent (Thermo Fisher Scientific, 15596026) using 1mL syringe and 21G needle and RNA was purified from lysates according to the Phasemaker tubes protocol (Thermo Fisher Scientific, A33248). For cDNA synthesis, a 4-μg aliquot of total RNA was treated with TurboDNAse using the TurboDNA-free kit (Thermo Fisher Scientific, AM1907). cDNA was produced by mixing 10μL of DNAse-treated RNA with 4μL of 2.5 mM dNTP and 2 μL of 80μM Random Primer 9 (NEB, S1254S) or 100μM oligo-dT(15–18) (Integrated DNA Technologies), heating at 70°C for 10 min and cooling on ice. Subsequently, 2μL of M-MuLV buffer (NEB, M0253S), 0.25μL of Protector RNAse Inhibitor (Roche, 03335399001), 0.25μL of M-MuLV reverse transcriptase (NEB, M0253S) and 1.6μL of water were added followed by incubation at 42°C for 1 hour and 10 min at 90°C. To amplify p53 cDNA fragments, we used p53cDNA primers (S1 Table) to run the PCR on odT-based cDNA using Q5 High-Fidelity 2X Master Mix (NEB, M0492) with its standard program and annealing temperature of 64°C.

### Quantitative real-time polymerase chain reactions

Quantitative real-time polymerase chain reactions (qPCR) were prepared by adding 10μL of BrightGreen 2X qPCR MasterMix-No Dye (ABM, MasterMix-S), 2μL of 2.5μM primer mix, and 6μL of diluted cDNA samples (dilutions from 40 times to 640 times). qPCR was carried out using the QuantStudio 3 Real-Time PCR System (Thermo Fisher) or CFX96 (Bio-Rad) as follows: 95°C for 10 minutes, (95°C 30 sec, 60°C for 30 sec, 72°C for 30 sec) x 40 with primers listed in S1 Table. C_T_ (cycles to threshold) values were extracted from the raw data and analyzed in Microsoft Excel and R. For the p53-induction experiments (CPT treatment), fold changes were calculated using standard ΔΔC_T_ procedures, normalizing target gene data by *eef1a1a* (*ef1a*) and 18S rRNA gene values as well as by those of control (DMSO-treated) samples. The other qPCR experiments were normalized by beta-actin (*bactin*) and ef1a-new assays (S1 Table).

### Cloning and application of the miR-34a sensor vector

miR-34a reporter vector with EGFP and 3 antisense miR-34a sites was constructed from pCS2+EGFP-TA (Addgene, 49338) by inserting 3 miR-34a target sites (ACAACCAGCTAAGACACTGCCA) separated by TAGTA spacers. A 2μg aliquot of each miR34a-3xPT oligo (S1 Table) were annealed in 50μL reaction in annealing buffer (10mM Tris pH = 7.5, 50mM NaCl, 1mM EDTA). The annealing sample was heated at 95°C for 10 minutes and cooled to room temperature over 45 minutes. Next, 8μL of the annealed insert was phosphorylated by T4-polynucleotide kinase (NEB, M0201S) in a 10μL reaction at 37°C for 30 minutes and 65°C for 20 minutes. pCS2+EGFP-TA was digested with XhoI (NEB, R0146S) and XbaI (NEB, R0145S) and dephosphorylated with CIP (NEB, M0290S). pCS2+EGFP-TA XhoI-XbaI fragment was ligated with miR34a-3xPT insert using T4 DNA Ligase (NEB, M0202S) overnight at 16°C. The ligations were transformed into DH5α competent cells (Thermo Fisher Scientific, 18265017) and the resulting mini preps were purified using the QIAprep Spin Miniprep Kit (Qiagen, 27106) and sequenced (Genewiz). A correct bacterial clone of the pCS2+EGFP-miR34a-3xPT plasmid was grown for a midi-prep of this plasmid using ZymoPURE II Plasmid Midiprep Kit (Zymo Research, D4200).

### *In vitro* transcription for mRNA expression

Plasmid DNAs for most mRNA expression vectors were linearized with NotI overnight, extracted with Phenol:Chloroform:Isoamyl Alcohol (25:24:1) (Thermo Fisher, 15593031) in the Phase Lock Light 1.5 ml tubes and precipitated with ethanol, sodium acetate with added glycogen [41]. Cas9 mRNA was made from pT3TS-nCas9n plasmid [42] (Addgene, 46757) after its linearization with XbaI and purifications as above. mRNAs were then synthesized using mMESSAGE mMACHINE T3 Transcription Kit (Thermo Fisher Scientific, AM1348) and purified by LiCl precipitation according to the manufacturer’s instructions.

### Whole-mount *in situ* hybridization

The genomic *miR-34a* probe corresponds to the 0.44 kb genomic region around the *miR-34a* primary transcript, a probe design based on a previous published protocol [43]. The probe template was amplified from genomic DNA with *mir-34a_ISH_for* and *T7-mir-34a_ISH_rev* primers (S1 Table) using *Taq* DNA polymerase according to the program: 95°C for 3 min; (95°C for 30 s, 52°C for 30 s, 72°C for 30 s) x 36 cycles; 72°C for 1 min. The templates for the *alas2* and *myb* anti-sense RNA probes were amplified with their respective primers (S1 Table) from the pooled wild-type embryo cDNA using Q5 High-Fidelity 2X Master Mix (NEB, M0492) at 65°C annealing temperature. The probes were synthesized from the template PCR product by DIG RNA Labeling Kit (SP6/T7) (Roche, 11175025910) according to the kit instructions. Whole-mount *in situ* hybridization (WMISH) was carried out according to the protocol by Lauter *et al*. [44] except that the detection step was performed using Anti-Digoxigenin-AP, Fab fragments (Roche, 11093274910) at 1:2500 dilution in the blocking buffer and the staining step was done with BCIP/NBT Alkaline Phosphatase (AP) Substrate Kit (Vector Laboratories, SK-5400) according to the kit instructions. The stained embryos were then fixed in 4% PFA for 30 minutes, washed in PBST and embedded into 80% glycerol for imaging.

### CRISPR/Cas9 deletion *miR-34a* mutant generation and *tp53* mutant genotyping

The *miR-34a* gene was deleted using 6 single guide RNA (sgRNA) (3 on each side of the gene). Oligos containing T7 promoter, sgRNA spacer and a scaffold overlap region were synthesized (S1 Table). sgRNAs were generated by performing an overlap-extension PCR of the sense sgRNA oligos each combined with *Rev_sgRNA_scaffold* oligo. sgRNA template synthesis reactions were set up using *Taq* DNA polymerase (ABM, G009) by combining 10μL of 10× buffer, 6μL of 25mM MgSO_4_, 2μL of 10mM dNTP, 5μL of each oligo at 25μM, 71μL water and 1.5μL of Taq. The PCRs were run with a short program: 94°C for 5 min; 5 cycles: 94°C for 30s, 55°C for 30s, 72°C for 30s. The resulting PCR products were purified using QIAGEN Gel Extraction kit (QIAGEN, 28704) and used for *in vitro* transcription using MEGAshortscript T7 kit (Thermo Fisher Scientific, AM1354). The sgRNAs were purified according to the kit instructions. Eggs were injected with Cas9 mRNA (250 ng/μL) and a mix of sgRNAs (300 ng/μL). The genotyping of both embryos and adults was performed by first preparing DNA samples for PCR as described previously [45]. The PCRs were then run with *miR-34a_assay_for* and *miR-34a_assay_rev* primers (S1 Table) using *Taq* DNA polymerase according to the touch-down PCR (tdPCR) method: 94°C for 3 min; **10 cycles**: 94°C for 30 s, *61*°C (with 1°C decrease every cycle), 72°C for 30s, **25 cycles**: 94°C for 30s, *51*°C, 72°C for 30s. The resulting PCR products for the wild-type allele (532 bp) and mutant allele (296 bp) were visualized by standard agarose gel electrophoresis. To genotype *tp53* deletion mutants we performed tdPCR at 55°C with *p53_null* and *R217_SA* primers to detect the null and wild-type alleles, respectively. Detection of the R217H allele was done by *R217_SA* PCR as just described followed by MspI (NEB, R0106S) digestion [34], where the lack of digestion indicates a wild-type genotype and near-complete digestion signifies a homozygous mutant genotype.

### Overexpression of miR-34a mimic

Transient miR-34a overexpression was achieved via injection of miRIDIAN microRNA Mimic for hsa-miR-34a-5p (Horizon Discovery) into wild-type zebrafish embryos at the one-cell stage. As a control, we used miRIDIAN microRNA Mimic Negative Control #1 (Horizon Discovery). The sequence of this *microRNA* mimic is identical to the zebrafish *miR-34a* thus ensuring the specificity. Both control and *miR-34a* mimics were injected at 10μM concentration. To assay the activity of the miR-34a mimic, we co-injected mRNAs of EGFP-3xmiR-34a_site-pA or EGFP-pA (45ng/μL) and TagRFP-pA (75 ng/μL), up to a final volume of 5μL. Fish were then incubated at 28°C until imaging at 16 hpf or 30 hpf. Injected embryos injected were imaged using ZEISS Axio Zoom.V16 microscope for green and red fluorescence.

### Mature miR-34a quantitative PCR analysis

Trizol-purified total RNA was used for analysis of mature miR-34a. Mature miR-34a analysis was performed after cDNA synthesis with stem-loop oligo (miR-34a_SL_RT) and U6 RNA-specific oligo (U6_RT_primer) (S1 Table) according to the following procedure: 10μL reaction contained 5.5μL of total RNA (up to 5μg of RNA), 1μL of 5μM miR-34a_SL_RT, 1μL of 5μM U6_RT_primer, 1μL of 10 mM dNTP, 1μL of 10x M-MuLV buffer, 0.25μL Protector RNAse Inhibitor, 0.25 μL M-MuLV reverse transcriptase. The reactions were incubated at 25°C for 5 min, and at 42°C for 60 min and inactivated at 70°C for 5 min. The reactions were then diluted 1:20 with water and used in qPCR analysis using the mature miR-34a assay (miR-34a_mature_o2_for (overlaps the stem-loop oligo by 2 nt) and Rev_SL_qPCR_rev) and U6 assay (U6_for and U6_rev) (S1 Table). The 10μL qPCR reaction was set up as follows: 5μL of 2X BlasTaq qPCR MasterMix (ABM, G891), 1μL of 1μM forward and reverse primer mix and 4μL of cDNA solution and run according to this program: 95°C for 3 min, followed by 40 cycles of 95°C for 10s, 60°C for 35s. Assay specificity was established using serial dilutions of cDNA, noRT controls, water controls and cDNA samples from *miR-34a-/-* zebrafish embryos.

### RNA sequencing (RNA-Seq) procedures and data analysis

We extracted RNA as described above from miR-34a-/- and wild-type embryos, both of which are in the *casper* genetic background [46], at 8, 28 and 72 hpf stages in pools of 30. Total RNA samples were measured and analyzed for integrity on Agilent Tape Station. Samples with RNA integration number ≥8 were selected for library preparation. Poly-A enrichment was performed from 20 μg of total RNA to enrich for mRNA (Dynabeads mRNA DIRECT Micro Kit, Thermo Fisher). 100ng of poly-A enriched RNA was fragmented using RNase III and purified using magnetic bead clean up module (RNA Seq V2 kit, Life Technologies). The size distribution of the fragmented RNA was assessed on Agilent Tape Station using RNA HS screen tape assay and 50ng of fragmented polyA-enriched RNA was used to prepare whole transcriptome library (RNA seq V2). Yield and size distribution of the library was analyzed on Agilent Tape Station using D1000 screen tape. Barcoded library was equally pooled and amplified onto Ion Sphere Particles (ISPs) from Ion Pi HiQ OT2 kit (Life Technologies). ISPs enriched with template library were loaded onto Ion PI chip V3 and sequenced on Ion Proton from Thermo Fisher. The 8 and 72 hpf samples were sequenced by the Stem Core Sequencing Facility (Ottawa Hospital Research Institute) using NextSeq 500. The raw reads had the adapters removed and filtered by the quality of 20 with the Trim Galore (v0.4.4) (Krueger F, Trimgalore (2021), https://github.com/FelixKrueger/TrimGalore). The RNA-Seq data were mapped with STAR (v2.7) [47] using the Genome Reference Consortium Zebrafish Build 11 (danRer11). Differentially expressed genes at each stage were identified using edgeR Bioconductor package [48] using the 2-fold up- or down-regulation thresholds and False-discovery rate ≤ 0.05 as parameters. Principal Component Analysis (PCA) and Multi-dimensional Scaling (MDS) plots were produced using DESeq2 package [49]. Heatmaps were produced using pheatmap package (https://cran.r-project.org/web/packages/pheatmap/index.html) after performing variance stabilizing transformation using a function from DESeq2 (https://bioconductor.org/packages/release/bioc/html/DESeq2.html). Heatmaps of the inferred p53 target genes were based on known p53 target genes from IARC (International Agency for Research on Cancer) mapped to the zebrafish orthologs. These p53 target orthologs were then intersected with differentially expressed genes from the RNA-seq dataset.

### Tumor watch setup, follow-up, and statistical analysis

We set up the tumor watch experiment by following tumor development in *tp53-/-*, *tp53*^*R217H/R217H*^ [34] and compound mutant *miR-34a-/-;tp53-/-* and *miR-34a-/-;tp53*^*R217H/R217H*^ adult zebrafish. The compound mutants were generated by first making double heterozygotes, breeding them to obtain double homozygotes and then mass breeding these animals to generate the indicated compound mutant lines (maternal-zygotic for both genes). The number of animals in the analyzed groups ranged from 24 to 59. From 4 to approximately 16 months, we examined all these animals for signs of tumor development and euthanized the tumor-bearing ones for histological analysis. The records of times when tumors developed in respective groups were then used for survival analysis using R statistical computing language with ‘survival’, ‘survminer’, ‘dplyr’ and ‘ggplot2’. The statistical significance was determined by the survdiff function implementing the log-rank or Mantel-Haenszel test with χ^2^ value used to determine the P-value of significance [50].

### Tumor histological analysis

Zebrafish were fixed in 10% neutral buffered formalin, bisected in the sagittal plane, and processed whole, using a Leica ASP6025S tissue processor. Fish were then embedded in Paraplast Surgiplast paraffin and sectioned on a Leica RM 2235 microtome. Slides were stained on a Somagen Tissue-Tek Prisma using standard hematoxylin and eosin and coverslipped by a Somagen Tissue-Tek Glas. Slides were examined by a board-certified pediatric and anatomical pathologist (C.M.) using a Nikon Eclipse Ni light microscope. Digital images were captured with a 5-megapixel Olympus SC50 microscope mounted camera using Olympus cellSens software.

### O-dianisidine staining and quantification

Embryos were collected and incubated in freshly made O-dianisidine staining solution (0.01M sodium acetate pH 4.5, 0.65% hydrogen peroxide, 40% ethanol, 0.6 mg/ml O-dianisidine (MilliporeSigma, D9143-5G)) for 15 minutes in the dark at 28°C. After washing in PBST, the embryos were fixed in 4% PFA overnight. Stained embryos were imaged with Zeiss SteREO Discovery V8. Pixel classification and signal intensity measurements were performed by ilastik (0.5) [51] (custom classifier training) and Cell Profiler (2.0.11710) [52] (classifier application to data) using the previously published pipeline [53].

## Results

### Conservation and expression of *miR-34a* and *miR-34b/miR-34c* genes in zebrafish

**Fig 1:**
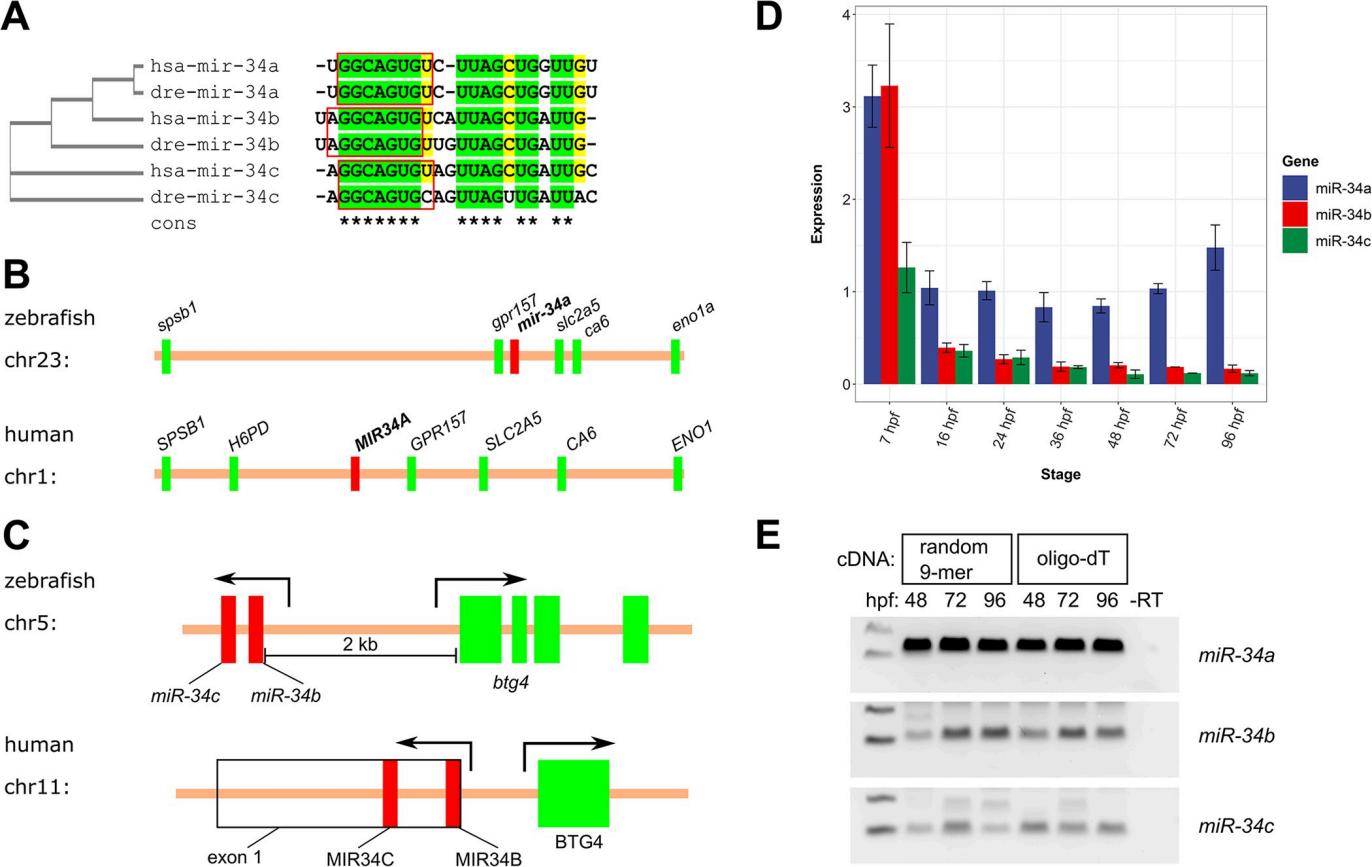
Conservation, genomic synteny and expression timing of microRNA-34 family members in zebrafish. **(A)** Multiple sequence alignment of human (hsa) and zebrafish (dre) microRNA-34 (a, b, c) sequences. Identical nucleotides are marked with “*” in the consensus and in green. Nearly identical nucleotides (in 5 out of 6 microRNAs) are highlighted in yellow. The seed sequences are surrounded by red boxes. A cladogram showing phylogenetic relatedness of these microRNAs is shown next to microRNA names. **(B)** Synteny of genomic miRNA-34a regions in zebrafish and human. Start positions of the genes are marked with small rectangles (red for miR-34a and green for all other genes). **(C)** Synteny of *miR-34b/c* cluster location next to the *btg4*/BTG4 gene in zebrafish and humans. The miR-34b and miR-34c primary transcripts in both species are indicated with red rectangles and *btg4*/BTG4 exons are shown in green. *MIR34B* and *MIR34C* transcripts in human consist of a single exon shown by a rectangle with a black outline. Promoter regions are indicated with directed arrows. The opposite orientation of the promoters for the *miR-34b/c* cluster and *btg4*/BTG4 gene is also conserved from zebrafish to humans. **(D)** Quantitative real-time PCR analysis of the relative expression of *miR-34a*,*b*,*c* at different stages of zebrafish development. Triplicate biological samples of each stage and duplicate technical replicates were used for the analysis. The expression levels were normalized using 18S rRNA expression and the relative levels of all samples and genes were calculated relative to the level of *miR-34a* at 24 hpf. **(E)** Agarose gel analysis of semi-quantitative RT-PCRs of *miR-34a*,*b*,*c* transcripts at 48, 72 and 96 hpf stage zebrafish cDNAs synthesized with either random 9-mer or oligo-dT oligos.

We first assessed the evolutionary conservation and expression of the *miR-34a* and *miR-34b/c* genes in zebrafish. All the miR-34s are very similar to each other and to their human homologs, with miR-34a being identical, and miR-34b and miR-34c having two and three differences from their human homologs, respectively, and all of them having nearly identical seed sequences (positions 2–7) important for miRNA binding (Fig 1A). *miR-34a* is also highly syntenic with the human *MIR34A* gene with one inversion among the neighboring genes (Fig 1B). The zebrafish *miR-34b*/*c* and *btg4* genes face in opposite orientations similar to the human genome (Fig 1C). Quantitative real-time PCR (qPCR) assays for the primary transcripts of all 3 miR-34s were used to study the relative expression levels of *miR-34* genes during development. The highest relative levels for all three *miR-34* transcripts occurred at 7 hpf (Fig 1D). At later stages (16, 24, 36, 48, 72 and 96 hpf), the relative expression levels of all miR-34s were lower than at 7 hpf, but *miR-34a* expression remained stable, whereas *miR-34b* and *miR-34c* expression decreased further (Fig 1D). We also confirmed that *miR-34* genes were expressed from poly-adenylated transcripts since their primary transcripts could be amplified from both oligo-dT and random 9-mer cDNA (Fig 1E).

### Zebrafish miR-34 genes are regulated like p53 targets

**Fig 2:**
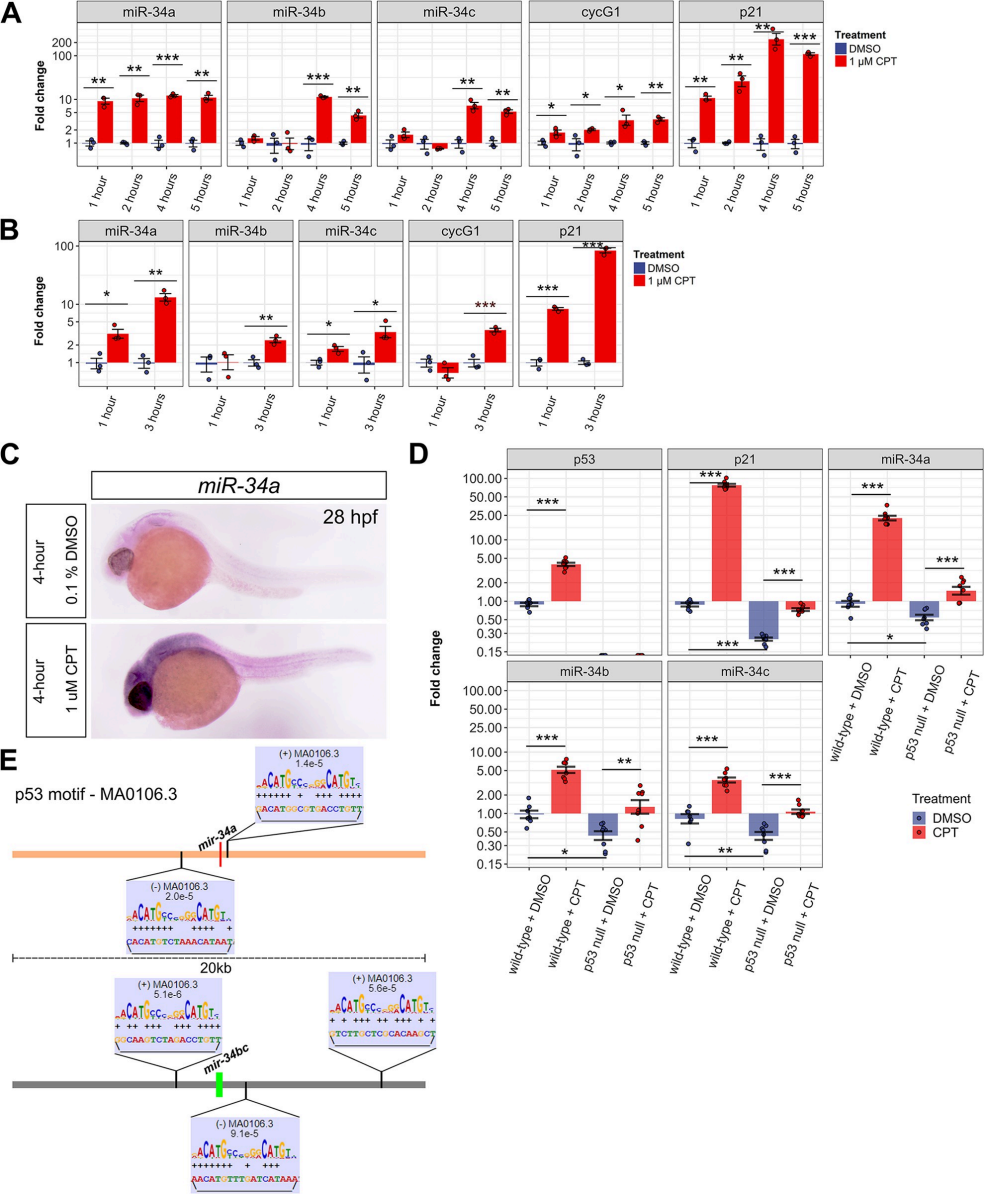
p53 induces all miR-34 genes in zebrafish but with different kinetics. (**A**) Quantitative PCR (qPCR) analysis of miR-34 genes (*miR-34a*, *miR-34b*, *miR-34c*) as well as p53 target genes *p21* and *cycG1* after 1, 2, 4 and 5 hours of treatments at 24 hpf with 0.1% DMSO or 1μM camptothecin (CPT), which induces DNA damage and p53 activation. (**B**) qPCR analysis of miR-34 genes as well as p53 target genes p21 and cycG1 after 1 and 3 hours of treatments at 48 hpf with 0.1% DMSO or 1 μM CPT. (**A, B**) The qPCR experiment was run with 4 biological replicates and 2 technical replicates. (**C**) Whole mount *in situ* hybridization analysis of *miR-34a* expression pattern after 4-hour treatment with DMSO or CPT in wild-type zebrafish embryos. Representative images of 40 embryos stained per condition are shown. (**D**) qPCR for p53 target genes (*p53*, *p21*) and *miR-34* genes in wild-type and *p53* null mutants after a 4-hour treatment at 24 hpf with either 0.1% DMSO or 1μM CPT. The qPCR experiment was run with 7 or 8 biological replicates and 2 technical replicates. Fold changes for each gene are indicated on the log10-scaled y-axis relative to control. Standard errors are used for error bars. Significantly different genes in (**A**, **B, D**) are indicated by ‘***’ (P-value < 0.001), ‘**’ (P-value < 0.01) and ‘*’ (P-value < 0.05). (**E**) Positions and alignments of the p53 transcription factor motif in the 20-kb regions around the *miR-34a* and *miR-34b/c* genes performed using the MAST software. The alignment figure position above or below the genomic sequence line indicates the strand (+ or -, respectively). The P-values for the motif matches are indicated on the alignment inserts.

We next examined inducibility of *miR-34* genes by p53 by performing a time-course experiment on 24 hpf zebrafish embryos by treating them for 1, 2, 4 and 5 hours with 1μM CPT or 0.1% DMSO as a control, and analyzed *miR-34* and *cycG1* and *p21*, p53 target gene transcripts. *miR-34a*, *cycG1* and *p21* genes were rapidly induced following 1-hour treatment and their higher expression was maintained during longer treatments, whereas *miR-34b* and *miR-34c* were induced only after 4 hours of CPT exposure (Fig 2A). When the same CPT treatment was performed on 48 hpf embryos for 1 or 3 hours, *miR-34a*, *miR-34c* and *p21* were induced following 1-hour exposure and all target genes analyzed were induced after the 3-hour treatment (Fig 2B). We also aimed to determine the tissue specificity of *miR-34a* gene induction after 4-hour 1μM CPT treatment compared to controls by WMISH at 28 hpf and observed global induction of its expression (Fig 2C). To support the idea that *miR-34* genes are p53 targets, we performed 1μM CPT treatment in both wild-type and *tp53*^*-/-*^ mutants [33] for 4 hours. p53 loss indeed partially reduced the baseline levels of *p21*, *miR-34a*, *miR-34b* and *miR-34c* (Fig 2D). The induction of *p21* and *miR-34* genes by CPT treatment was significantly reduced in the *tp53*^*-/-*^ fish, strongly indicating that *miR-34* genes are p53 targets in the zebrafish. We also identified highly significant adjacent matches of the p53 transcription factor motif on both sides of *miR-34a* and *miR-34b/c* genes when looking at the 20-kb regions around these genes (Fig 2E and S2 Table). These putative p53 binding sites are analogous to what was reported for human miR-34 genes [9,11,12], but the p53 motifs are much closer to the exons encoding miR-34s in zebrafish than in humans since human *miR-34* genes contain large introns.

### Loss of *miR-34a* in zebrafish does not lead to a morphologically distinguishable phenotype

To generate a *miR-34a* mutant p53 genetic modifier model, we employed 6 sgRNAs (not all of which were known to work *a priori*) in our CRISPR/Cas9 deletion strategy to excise the *miR-34a* transcript (98-bp) (S1A Fig). The resulting deletions were later assayed using a PCR assay amplifying the target genomic region and the deletion products were readily detected in 14 out of 16 analyzed injected embryos (S1B Fig). Breeding injected fish followed by genotyping and sequencing this locus in F1 progeny (S1C and S1D Fig) identified animals with a 236-bp deletion allele, which were then bred to establish the *miR-34a* mutant line. Maternal-zygotic *miR-34a*-/- fish were morphologically normal, fertile and were used for subsequent experiments. To exclude *miR-34b*/c gene compensation for miR-34a loss, we performed a qPCR experiment for all 3 *miR-34* homologs, which confirmed loss of *miR-34a* in the mutant and no significant differences in *miR-34b* and *miR-34c* expression levels between wild-type and *miR-34a-/-* mutants (S1E Fig). Since apoptosis is a major function of p53 induction, we also tested if loss of miR-34a exerts any effect on p53-dependent apoptosis and found comparable levels of apoptosis in wild-type and *miR-34a*-/- embryos following 0.25, 0.5 and 1μM of CPT as determined qualitatively by acridine orange staining (S2 Fig).

### Gene expression profiling following camptothecin-induced DNA damage shows a minor contribution of *miR-34a*

**Fig 3:**
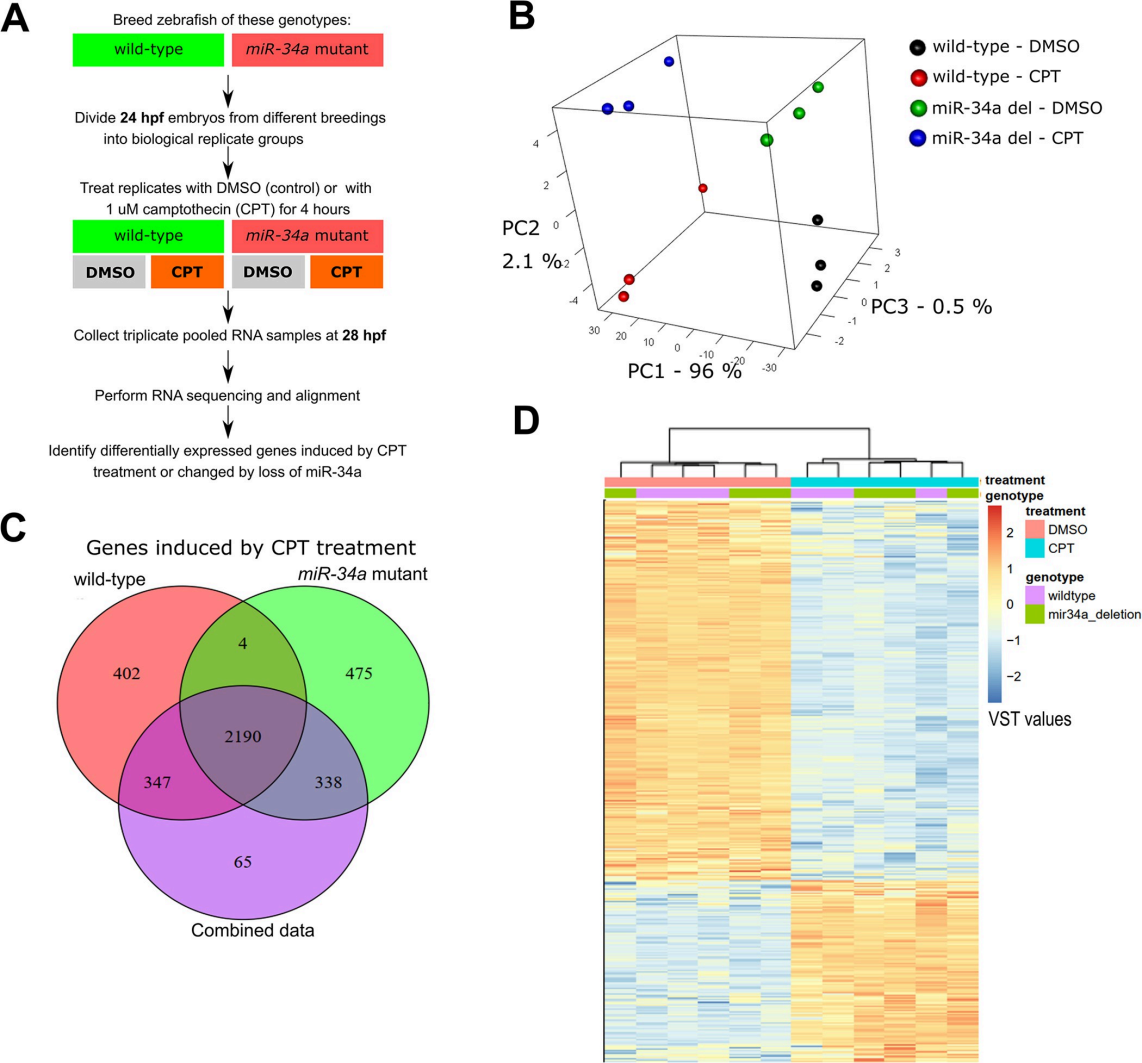
p53 activation by camptothecin treatment induces massive gene expression effects with a small contribution from *miR-34a*. **(A)** Experimental design of the RNA sequencing experiment to analyze how *miR-34a* loss affects gene expression under normal and DNA damage treatment conditions. **(B)** Principal component analysis of gene expression values in all RNA sequencing samples. **(C)** Venn diagram for differentially expressed genes (DEGs) (fold-change ≥ 2 and FDR ≤ 0.05 as cut-offs) in the combined dataset (both genotypes), wild-type and *miR-34a* mutant subsets. **(D)** Hierarchical clustering of variance-stabilized transformed normalized counts (VST values) for all DEGs based on the treatment factor in full dataset. Treatment and genotype assignments are indicated at the top of the gene expression heatmap and the legend for the heatmap is shown to the right.

We then determined the gene expression changes by CPT treatment and if they were affected by miR-34a. Wild-type and *miR-34a-/-* embryos at 24 hpf were independently bred in triplicate, each divided into control and treatment groups and treated with either 0.005% DMSO or 1μM CPT in fish medium for 4 hours followed by RNA extraction and sequencing. (Fig 3A). Principal Component Analysis (PCA) of the complete expression data to determine sample clustering showed that the first component (PC1) explains 96% of variance and corresponds to treatment, whereas PC2 explains 2.1% of variance and separates samples by genotype (Fig 3B). Thus, while the DNA damage-induced effects dominate the observed gene expression profiles, lack of miR-34a has a small but significant effect. The total numbers of differentially expressed genes (DEGs) due to CPT treatment (fold-change ≥ 2 and false-discovery rate (FDR) ≤ 0.05) were 2940 DEGs in the combined dataset, 2943 DEGs in the wild-type and 3007 in the *miR-34a-/-* data subsets (S1 File), of which 2190 were common in all subsets (Fig 3C). In hierarchical clustering of this dataset, samples clustered based on the treatment consistent with the PCA results (Fig 3D). Down-regulated genes were about twice as frequent as up-regulated genes and accounted for 67.7% of all DEGs in the combined dataset (S1 File and Fig 3D).

### *miR-34a* mutants exhibit significant expression changes but a normal p53 target activation pattern when compared to wild-type zebrafish

**Fig 4:**
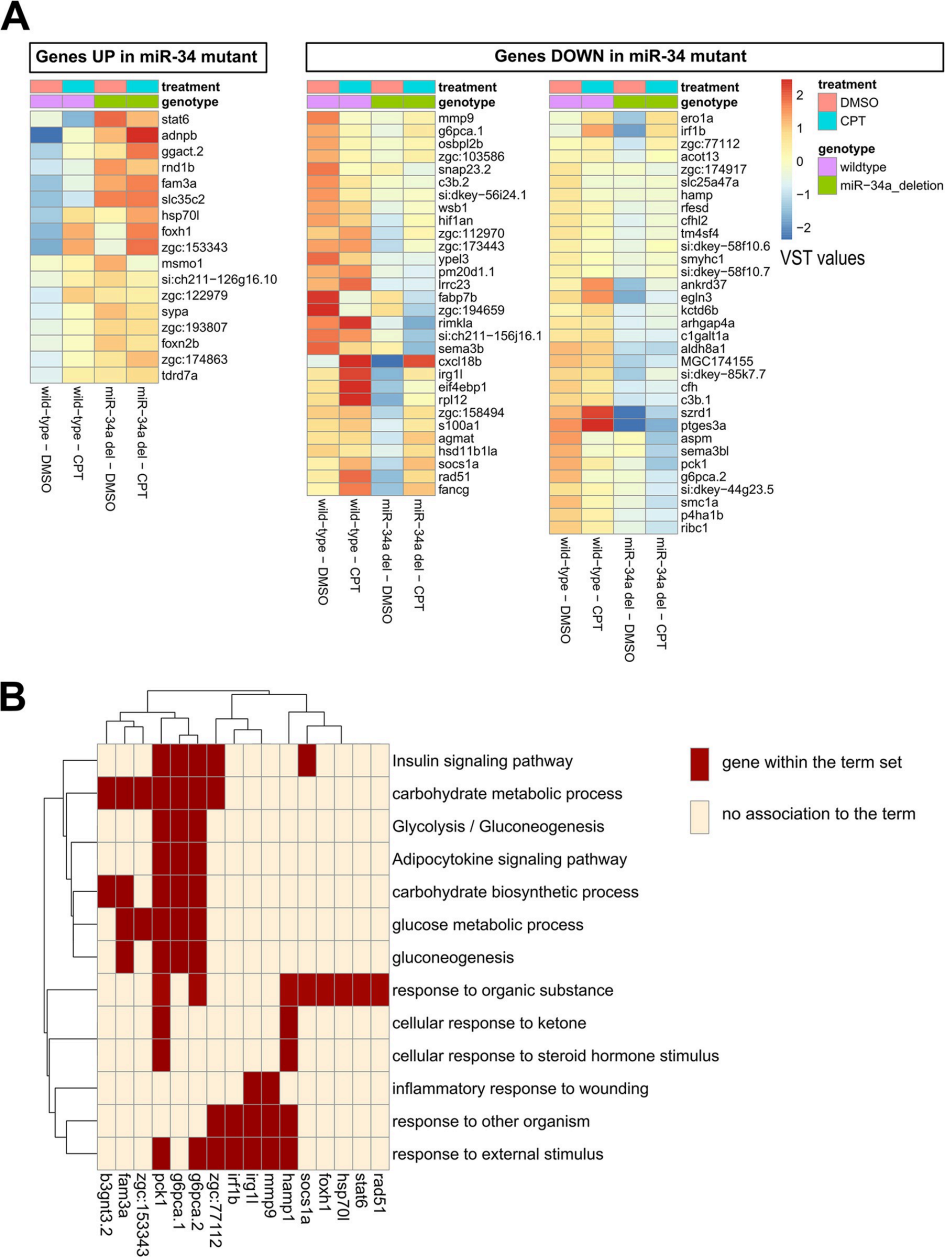
Expression changes due to loss of *miR-34a* identified by RNA-seq at 28 hpf. **(A)** Heatmap of variance-stabilized transformed normalized counts (VST values) in *miR-34a* deletion mutant relative to wild-type. The expression changes were determined by comparison of wild-type and *miR-34a* deletion mutant samples under DMSO-treated conditions and the heatmaps show all types of samples for completeness. **(B)** Clustering of Gene Ontology (GO) and KEGG terms as well as genes associated with them.

Although a microRNA knock-out may up-regulate many genes, the resulting perturbation of post-transcriptional regulation may lead to unexpected transcriptomic effects. When we compared 28 hpf *miR-34a-/-* and wild-type gene expression profiles, we identified only 19 up-regulated genes and 61 down-regulated genes (fold-change ≥ 2 and FDR ≤ 0.05; S2 File). Interestingly, the heatmap of the up-regulated genes in *miR-34a-/-* samples showed their up-regulation independent of treatment (Fig 4A). Some down-regulated genes in *miR-34a-/-* zebrafish also showed increased expression after CPT treatment while others did not change their expression apart from a decrease in *miR-34a-/-* samples (Fig 4A). GO BP and KEGG pathway enrichment tool application to the *miR-34a-/-* DEGs identified two clusters of enriched terms: carbohydrate metabolism and the responses to other organisms and external stimuli (Fig 4B and S3 File). Some genes (*pck1*, *g6pca*.*2*, *zgc*:*77112* and *socs1a*) belong to both sets of enriched terms, suggesting some linkage between them.

The term “gluconeogenesis” epitomizes carbohydrate metabolism and contains 4 genes in our dataset (*fam3a*, *pck1*, *g6pca*.*1*, *g6pca*.*2*), of which *fam3a* was up-regulated and the other 3 genes were down-regulated. This suggests a coordinated down-regulation of gluconeogenesis in *miR-34a-/-* embryos, since up-regulation of FAM3A in the mouse is known to suppress gluconeogenesis [54] and the same effect is known for downregulation of genes coding for the rate-limiting enzymes (*pck1*, *g6pca*.*1*, *g6pca*.*2*) [55]. The “response to organic substance” contains both up-regulated genes (*stat6*, *foxh1*, *hsp70l*) and down-regulated genes (*rad51*, *socs1a*, *pck1*, *g6pca*.*2*, *hamp1*), likely due to the general nature of the term. The “response” terms in Fig 4B only include down-regulated genes and at least 4 of them function in innate immunity (*irf1b*, *irg1l*, *mmp9*, *hamp1*), which implies an effect of *miR-34a* loss on immune function. Other notable expression changes include down-regulation of DNA repair genes (*rad51*, *fancg*) and a decrease in hypoxia-related genes (*hif1an*, *egln3*). Lacking known p53 targets among DEGs in the wild-type vs. *miR-34a-/-* post-CPT comparison, we visualized expression of zebrafish homologs of known p53 target genes (S4 File) and found that wild-type and *miR-34a-/-* samples have very similar levels of induction of these genes (S3 Fig), which was confirmed statistically.

### Compound *miR-34a* and *tp53* mutants show miR-34a contributions to tumor suppression

**Fig 5:**
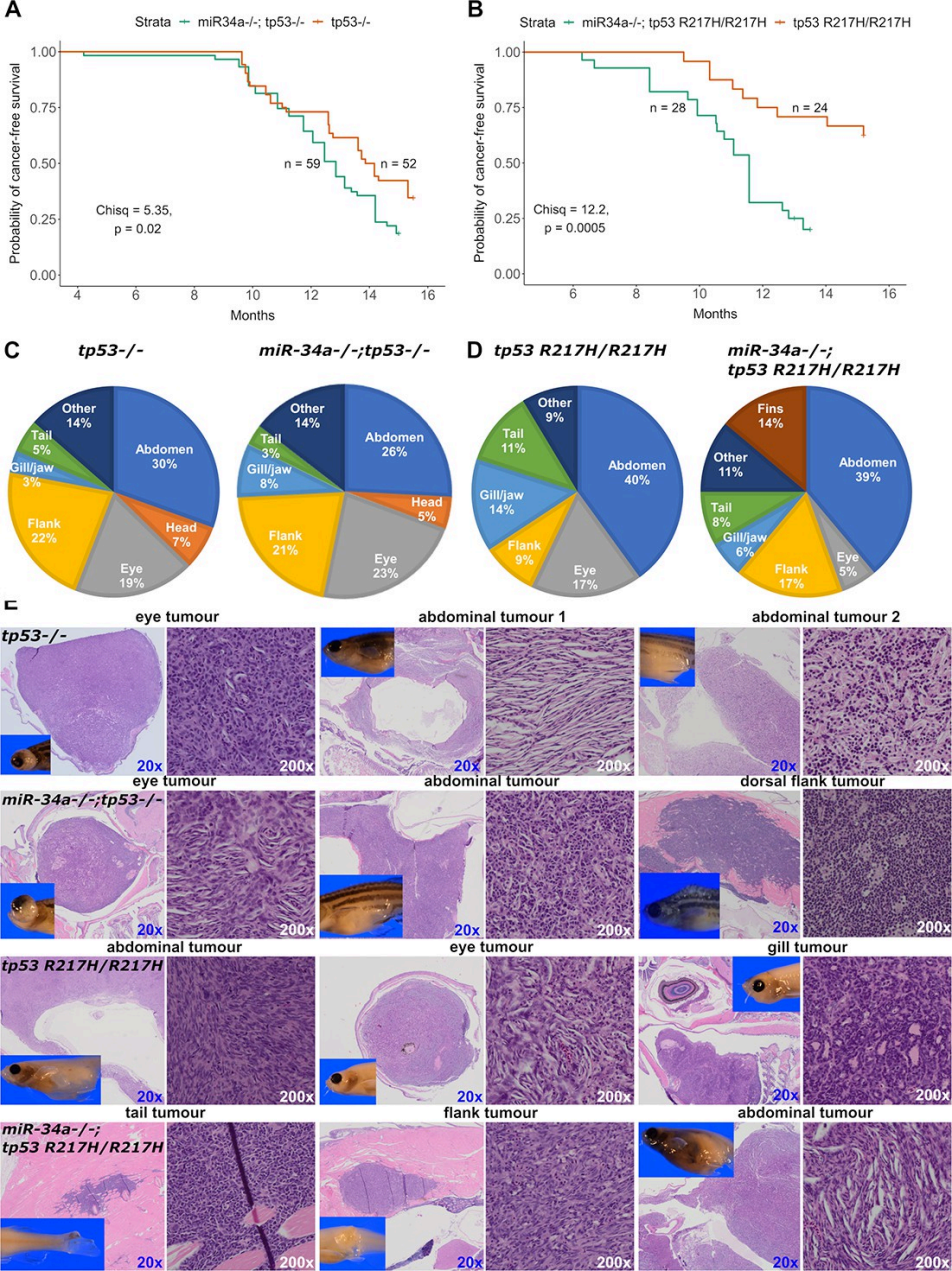
Tumor development due to *tp53* mutations in zebrafish is enhanced by loss of *miR-34a*. **(A)** Kaplan-Meier survival curves for *tp53-/-* and *miR-34a-/-;tp53-/-* zebrafish. (**B**) Kaplan-Meier survival curves for *tp53*^*R217H/R217H*^ and *miR-34a-/-;tp53*^*R217H/R217H*^ zebrafish. (**C, D**) Anatomical categorization of the tumors in zebrafish adults of the genotypes shown in (**A, B**). Broad anatomical categories are shown, which are not precisely related to the histological tumor types; ‘Other’ indicates that a fish had a cancer-related pathology such as a possible leukemia, but no visible tumor. (**E**) Hematoxylin-eosin staining of selected tumors identified during the tumor watch experiment. Each row shows 3 examples from the zebrafish of the indicated genotype on the first image in each row. For each tumor low (20x) and high-magnification (200x) images are shown as well as an inset of gross morphology of the zebrafish analyzed for tumor histology.

Despite *miR-34a* loss not affecting p53 target genes, we evaluated how miR-34a affects tumor development in p53 mutant zebrafish given that epigenetic silencing of the *MIR34A* gene in humans carrying *TP53* mutant alleles contributes to cancer progression [15]. We used *tp53-/-* and *tp53^R217H/R217H^* as control mutant lines and generated the homozygous maternal-zygotic *miR-34a-/-; tp53-/-* and *miR-34a-/-; tp53*^*R217H/R217H*^ compound mutant lines. We monitored visible tumor formation in control *tp53* mutants and in compound mutant lines on a weekly basis from 4 to 16 months and collected tumor-bearing fish for histology. The resulting tumor-onset curves (probability of tumor-free survival) show that *miR-34a-/-; tp53-/-* compound mutants exhibited a small but significant increase in the rate of tumor development compared to *tp53-/-* alone (Fig 5A). Interestingly, in the case of *miR-34a-/-;* *tp53^R217H/R217H^* compound mutants, *miR-34a* loss converted the mildly cancer-prone *tp53^R217H/R217H^* mutant to become as cancer-susceptible as either *tp53-/-* or *miR-34a-/-; tp53-/-* (Fig 5B). For each pair of genotypes, we also categorized the tumors by their anatomical location such as abdomen, head or eye (Fig 5C and 5D). The *miR-34a-/-; tp53*^*R217H/R217H*^ fish also had a significant fraction of animals with tumors in their fins. The “Other” category included sick fish with raised scales or a balloon-like appearance, but without a clearly defined tumor, which this is consistent with the previously published leukemic phenotype in *tp53-/-* zebrafish [33]. We then performed histological analysis of several tumors from each genotype: *tp53-/-* (n = 8), *miR-34a-/-; tp53-/-* (n = 9), *tp53*^*R217H/R217H*^ (n = 8), *miR-34a-/-; tp53*^*R217H/R217H*^ (n = 6). Two main histologies were observed in all the genotypes: spindle cell-like sarcomas and small blue round cell (SBRC) tumors (Fig 5E). Spindle cell-like sarcomas likely correspond to malignant peripheral nerve-sheath tumors (MPNSTs), whereas SBRC likely correspond to angiosarcomas and other sarcomas. Overall, *miR-34a* loss accelerates tumor development and may impact the tumor cell type spectrum and their anatomical location in compound mutants compared to the *tp53-/-* and *tp53*^*R217H/R217H*^ mutants.

To assess if *tp53* R217H transcript is indeed expressed in tumors of fish homozygous for this allele, we collected abdominal tumors from 2 *tp53*^*R217H/R217H*^ and 3 *miR-34a-/-; tp53*^*R217H/R217H*^ adults as well as one eye from each fish and extracted RNA from all these samples; 6 dpf wild-type and *miR-34a-/-; tp53*^*R217H/R217H*^ larval fish samples served as controls (S4A Fig). The expression of *tp53* was present in all samples but the tumor samples were not significantly different from the eyes, whereas most adult samples had higher tp53 levels than in the larval samples (S4B and S4C Fig).

### Expression profiling of wild-type vs *miR-34a-/-* mutants at 8 and 72 hpf shows a wide range of transcriptomic effects

**Fig 6:**
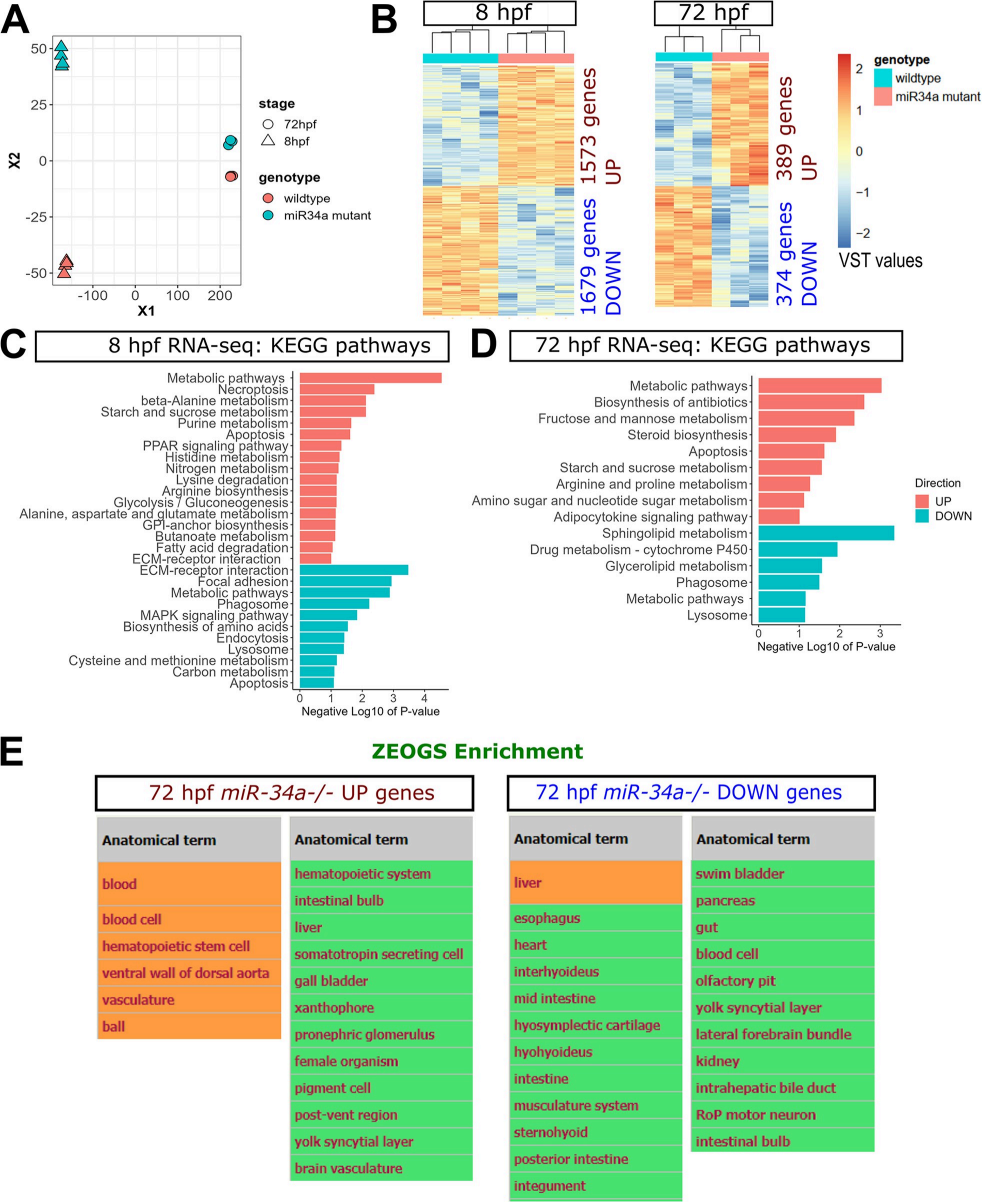
Expression profiling by RNA-seq of wild-type vs *miR-34a-/-* embryos at 8 and 72 hpf. **(A)** Multi-dimensional scaling (MDS) 2-dimensional projection of RNA-seq count matrices for all samples. **(B)** Heatmaps of variance-stabilized transformed normalized counts (VST values) for samples at both stages with the numbers of genes up-regulated (UP) and down-regulated (DOWN) in *miR-34a-/-*. **(C)** KEGG pathway analysis for DEGs identified in the 8 hpf dataset. **(D)** KEGG pathway analysis for DEGs identified in the 72 hpf dataset. DEGs in 8 hpf and 72 hpf datasets were defined with fold-change ≥ 2 and FDR ≤ 0.05 as cut-offs. **(E)** REVIGO plot of Gene Ontology Biological Process terms identified for up-regulated genes in the 72 hpf dataset. **(F)** REVIGO plot of Gene Ontology Biological Process terms identified for down-regulated genes in the 72 hpf dataset. **(G)** Anatomical term enrichment using Zebrafish Expression Ontology of Gene Sets (ZEOGS) of both UP and DOWN-regulated genes in the 72 hpf dataset. Orange color indicates FDR q-value < 0.1 significance after Benjamini-Hochberg correction and green indicates P-value < 0.1 before correction.

Since only a small number of genes were affected by miR-34a loss at 28 hpf, we performed further RNA-seq experiments comparing wild-type and *miR-34a-/-* mutants at earlier and later timepoints (8 and 72 hpf) to determine the expression changes from specific *miR-34a* inactivation and to compare them with the previously reported knock-down data [26]. Dimension reduction showed much greater wild-type versus mutant differences at 8 hpf than at 72 hpf (Fig 6A). Although we meticulously staged embryos based on morphology and fertilization timing, it is possible that development proceeds somewhat differently in the *miR-34a* mutant. Despite this caveat, it is likely that the high relative level of miR-34a in the wild-type embryos at 8 hpf led to larger transcriptomic effects (1573 genes UP and 1679 genes DOWN) than at 72 hpf (389 genes UP and 374 genes DOWN) as defined using fold-change > 1.5 and FDR < 0.05 as cut-offs in both cases. Lacking systematically verified miR-34 target genes in zebrafish, we obtained predicted zebrafish miR-34 target genes from TargetScanFish (http://www.targetscan.org/fish_62/) [56] (S4 File) and determined their overlap with up-regulated genes in *miR-34a-/-* genes at either 8 or 72 hpf. In both cases, the overlaps were not significant at either a 1.5 or 2-fold change threshold. This suggests that the direct transcriptomic effects from *miR-34a* loss are either very weak, occur mainly at the translational level or require more refined methods of detection. Analysis of DEGs at 8 hpf using the DAVID tool [36] for GO BP and KEGG pathway term enrichment separately for UP and DOWN genes revealed several interesting groups of terms (S5 File, and Figs 6C and S5). For UP-regulated genes, transcription regulation genes were the most highly enriched. Interestingly, several UP genes were associated with germ cell development: “spermatogenesis”, “binding of sperm to zona pellucida”, “P granule organization”, “germ cell development” and “oogenesis”. This result is likely very significant since enhanced sperm production was observed in another zebrafish *miR-34a* mutant [28] and germ-cell expressed *tdrd7a* was increased in 8 and 28 hpf *miR-34a-/-* embryos. Importantly, up-regulated *tdrd5* is a predicted miR-34a target in zebrafish (S4 File). Thus, these expression changes could explain the increased sperm phenotype. The metabolism terms such as “lipid metabolic process”, “nucleotide metabolic process”, “glucose metabolism”, “respiratory chain”, and “drug catabolism” were the second largest group of GO BP terms. Metabolism terms also dominated the KEGG pathway enrichments, including amino acid metabolic terms, purine metabolism, glycolysis/gluconeogenesis, fatty acid degradation, glycosylphosphatidylinositol (GPI)-anchor biosynthesis and butanoate metabolism (Fig 6C). The “PPAR signaling pathway” KEGG term was also enriched, which is very significant since miR-34a regulates this pathway [57–59]. Together, these observations suggest that loss of *miR-34a* reprograms a significant part of the metabolic network. Cell adhesion, cytoskeleton-related and primarily integrin genes were also enriched among the UP DEGs suggesting an increased migration capability during development. Finally, two KEGG cell-death related terms were also enriched: apoptosis and necroptosis, which is highly relevant given the role of miR-34a in the regulation of cell survival.

The 1679 down-regulated DEGs at 8 hpf resulted in 11 KEGG pathways and 58 GO BP term enrichments (S5 File). Metabolism and transcription regulation genes were the most prevalent among the DOWN genes. KEGG Pathway enrichment also revealed significant expression decreases among the genes associated with endocytosis, lysosome and phagosome as well as extracellular matrix interactions and apoptosis terms (Fig 6C). The GO BP terms for DOWN DEGs also included multiple developmental terms, regulation of cell cycle, signal transduction, cell migration and transport terms (S5 File and S5 Fig).

We also performed expression profiling at 72 hpf on whole wild-type and *miR-34a-/-* embryos. Since the whole-embryo RNA-seq includes RNA from multiple tissues, we determined which tissues contributed most to the DEGs identified in this dataset using Zebrafish Expression Ontology of Gene Sets (ZEOGS: http://zeogs.freehostspace.com/index.html), a specialized tool we developed based on the enrichment of anatomical terms associated with a subset of genes [60]. Blood, vasculature and hematopoiesis terms were the most enriched among the UP DEGs, suggesting enhanced hemoglobin expression. Other most enriched terms included liver, intestinal, kidney and pancreas associated most strongly with DOWN DEGs but also with UP DEGs, supporting the overall significant expression changes in these tissues (Fig 6E and S6 File). The musculature system, heart and cartilage terms were enriched only in the DOWN DEGs dataset, whereas pigment cell terms were present in the UP DEGs. Although miR-34a neural expression was previously reported [25], we identified only several nervous system terms of low enrichment (Fig 6E and S6 File), which could be due to whole-larval samples, limited expression changes in affected neurons and limited annotation of genes with anatomical terms.

Analysis of UP DEGs at 72 hpf using the DAVID tool showed a predominance of metabolism KEGG pathways, such as carbohydrate-related pathways, amino acid metabolism, adipocytokine pathway and apoptosis (S5 File and Fig 6D). To explore the gluconeogenesis pathway genes, we plotted their RNA-seq values at all three analyzed stages, finding that *pck1* was down-regulated at 28 and 72 hpf stages, *pck2* (the mitochondrial homolog of *pck1*) was unchanged, *fam3a* was up-regulated at 8 and 28 hpf stages, while *g6pca*.*1* and *g6pca*.*2* had different directions of regulation at different stages (S6 Fig). These results indicate that loss of miR-34a does not exert a fixed set of regulatory changes on gluconeogenesis genes.

GO biological process term enrichment revealed a strong enrichment of globin and hematopoiesis related genes, as well transcription factors and signal transduction genes (S5 File and S5 Fig). This pattern suggested enhanced erythrocyte production. Genes involved in potassium transport were also upregulated by loss of *miR-34a*. DOWN DEGs at 72 hpf had a different set of enriched KEGG metabolic pathways such as sphingolipid and glycerolipid metabolism, cytochrome P450, phagosome and lysosome suggesting a reduction in aerobic metabolic pathways (S5 File and Fig 6D). GO BP analysis for DOWN DEGs at 72 hpf provides additional details of gene expression changes (S5 File and S5 Fig) consistent with KEGG results. Proteolysis, oligosaccharide synthesis, peptide cross-linking genes and amino acid catabolism genes were reduced in expression. Ceramide biosynthesis genes, a part of sphingolipid metabolism, the cell-environment terms, such as signal transduction, transport, cell-matrix, and neuron cell-cell adhesion were likewise enriched among the DOWN DEGs. Angiogenesis and overlapping bacterial defense terms were also enriched among the down-regulated genes. In sum, *miR-34a* loss results in a complex set of metabolic, cell biological and regulatory gene expression changes, some of which may underlie tumor-enhancing effects of *miR-34a* loss.

### Up-regulation of erythrocyte and stem cell markers in *miR-34a-/-* mutant zebrafish larvae

**Fig 7:**
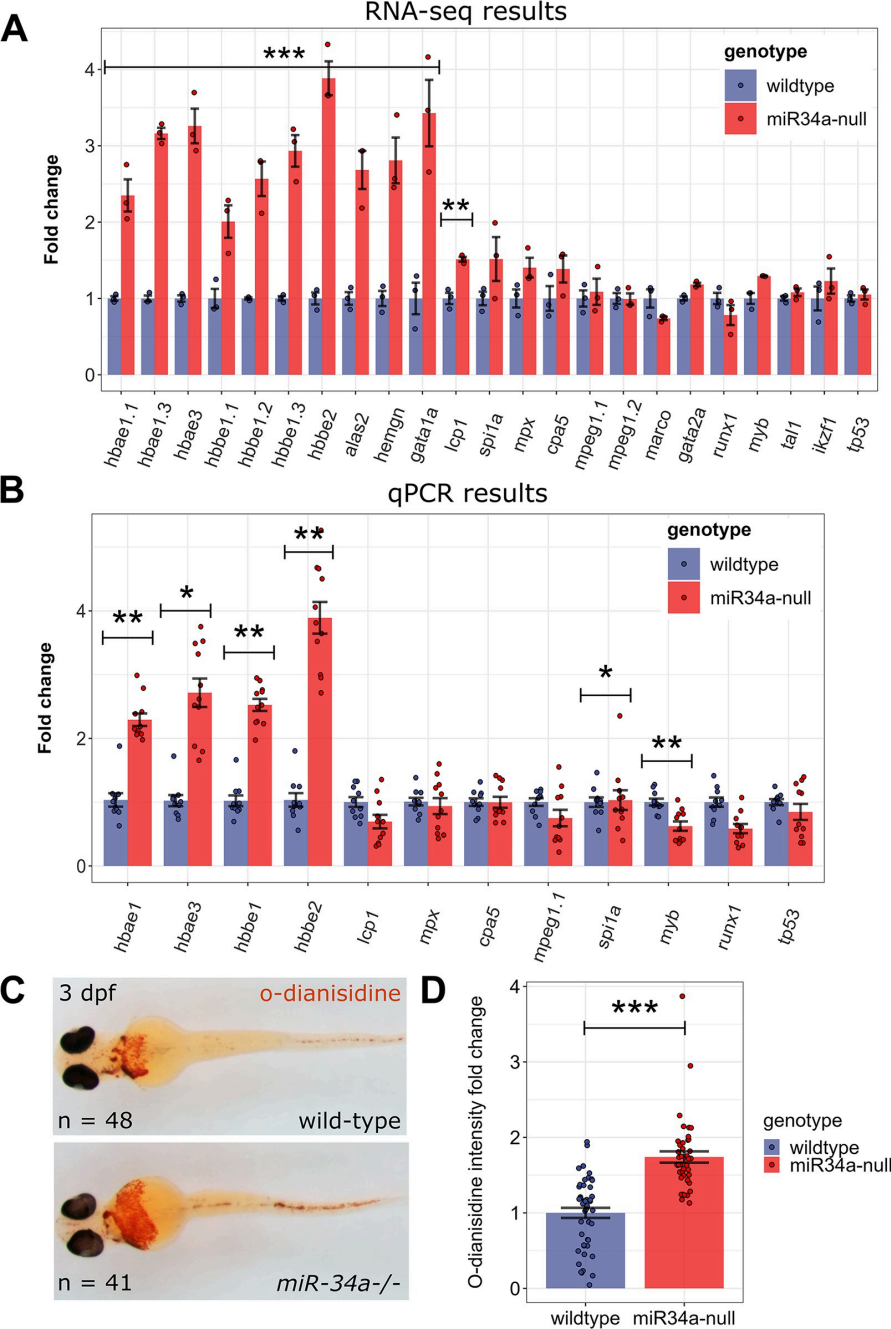
Analysis of blood cell type markers in 3 dpf wild-type and *miR-34a*-/- mutants reveals erythrocyte up-regulation in the mutants. **(A)** Bar graph of normalized relative expression values of blood-related genes and *tp53* in the 72 hpf RNA-seq dataset comprising data on 3 wild-type and *miR-34a*^-/-^ RNA samples. (**B**) qPCR verification of the selected blood cell type markers on 3dpf wild-type and *miR-34a*^-/-^ RNA samples (n = 10 and 11, respectively). Significantly different genes in (**A**) and (**B**) determined by a two-sample t-test with multiple-testing adjustment are indicated by ‘***’ (P-value < 0.001), ‘**’ (P-value < 0.01) and ‘*’ (P-value < 0.05). (**C**) Representative ventral images of o-dianisidine stained embryos at 3 dpf of both genotypes. The numbers of larvae are indicated. The staining was performed on embryos from two independent samples. (**D**) Quantification of o-dianisidine staining on 3dpf wild-type and *miR-34a*^-/-^ larvae (n = 48 and 42, respectively) using the ilastik-Cell Profiler pixel classification approach. Relative values of “Mean Intensity after thresholding” positively classified pixels are shown. The significance of the differences between the genotypes was calculated by the two-sample t-test (***; P-value < 9.956e-07).

Given these RNA-seq findings, we pursued the impact of *mir-34a* loss on blood cell development. Plots of the normalized RNA-seq values at 3 dpf showed that the mature erythrocyte markers (hemoglobin genes: *alas2*, *hemgn*) and the progenitor marker (*gata1a*) were significantly up-regulated in *miR-34a-/-* samples (Fig 7A). Among markers of other cell types, only a general leukocyte marker *lcp1* was significantly up-regulated by 1.5-fold in *miR-34a-/-* mutants, whereas other myeloid markers (*spi1a*, *mpx*, *cpa5*) were not significantly different (Fig 7A). We then obtained 10 and 11 independent pooled RNA samples of 3 dpf wild-type and *miR-34a-/-* mutants, respectively, and ran a qPCR experiment for selected blood cell type markers. This experimental validation of RNA-seq results confirmed up-regulation of hemoglobin genes but not of other mature blood cell type markers (Fig 7B). Interestingly, transcription factor genes *myb* and *runx1* involved in hematopoietic stem cell development were downregulated in the qPCR dataset (Fig 7B). We then assessed if erythrocyte marker up-regulation leads to a detectable increase of red blood cells and/or their hemoglobin content by staining 3 dpf wild-type and *miR-34a-/-* mutant embryos for hemoglobin with o-dianisidine, which indeed showed a visually detectable expanded labeled cell population in most *miR-34a-/-* mutant embryos (Fig 7C). We quantified o-dianisidine labeling using the manually trained ilastik classifier and showed a significant increase in erythrocyte/hemoglobin levels in *miR-34a-/-* mutant embryos (Fig 7D). Since *alas2* is a crucial heme synthesis enzyme gene and a predicted miR-34a target, we evaluated its expression by WMISH in 3 dpf wild-type and *miR-34a-/-* mutant embryos. *miR-34a-/-* mutant embryos more frequently scored as having a ‘High’ level of *alas2* staining compared to wild-type, which was also much more pronounced than in the similarly scored wild-type embryos (S7A and S7B Fig). We quantified hematopoietic stem cells by *myb* WMISH on 3 dpf wild-type and *miR-34a-/-* mutant embryos (S7C Fig) using an ilastik classifier and determined that the visually detectable increase in stem-cell levels in *miR-34a-/-* mutant embryos was significant (S7D Fig), suggesting a tissue-specific up-regulation.

### Transient overexpression of miR-34a impacts zebrafish embryo resistance to DNA damage but does not rescue the hematopoietic stem cell level changes in *miR-34a-/-* mutants

**Fig 8:**
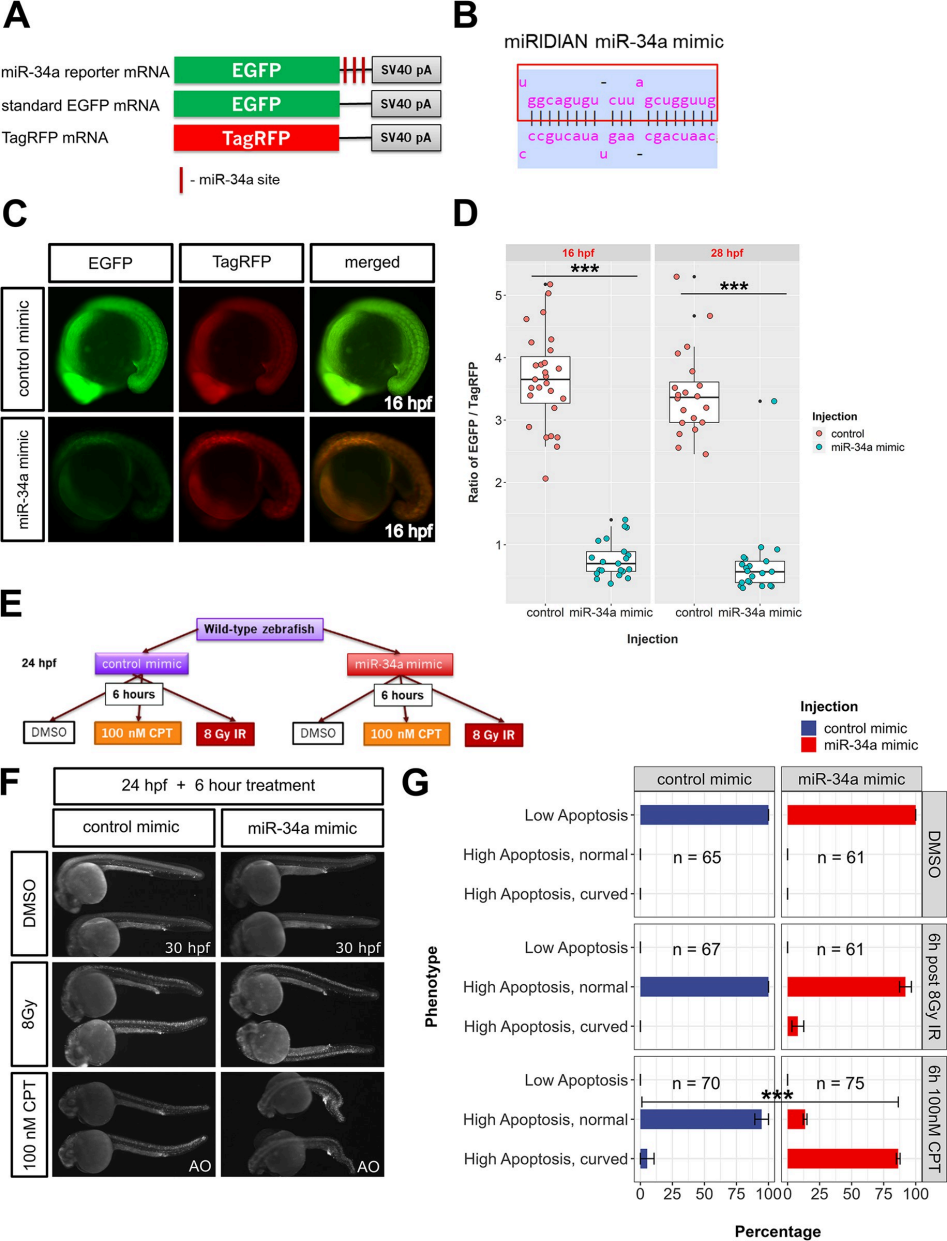
Transient overexpression of miR-34a sensitizes zebrafish embryos to camptothecin treatment. **(A)** mRNAs used for demonstrating miR-34a-mediated repression. miR-34a reporter RNA contains 3 miR-34a sites, which are anti-sense to the miR-34a sequence. EGFP mRNA is a control mRNA for the *miR-34a* reporter mRNA. TagRFP mRNA is used for normalizing injections. **(B)** Structure of the miRIDIAN miR-34a mimic. **(C)** Imaging of miR-34a reporter EGFP injected with a control or miR-34a mimic. **(D)** Quantification of EGFP/TagRFP signal ratios after control and *miR-34a* mimic injection at 16 and 28 hpf stages. Two-sample t-tests at both stages have P-values < 0.001 (***). **(E)** Experimental plan for testing how miR-34a overexpression affects the apoptotic phenotype after DNA damage by gamma-irradiation or by 100 nM camptothecin treatment. **(F)** Apoptosis and morphology imaging of zebrafish embryos at 30 hpf treated according to the plan in **(E)** using Acridine Orange (AO). **(G)** Quantification of the results of DNA damage treatments with a control or miR-34a mimic. Chi-square test on the 100 nM data produced P-value <10^−15^ as indicated (***).

miRNA loss-of-function and overexpression approaches provide complementary information about specific miRNA functions. miR-34a overexpression is physiologically relevant because of its induction by p53. Thus, artificial overexpression allows us to study what role *miR-34a* may play in the p53-mediated response when it is uncoupled from the other branches of this response. To perform transient overexpression of miR-34a, we developed a reporter system for visualizing the effects of a synthetic miR-34a on a target transcript, which consists of 3 mRNAs: EGFP mRNA (constitutive), EGFP-3x-miR-34a_site mRNA (reporter) and TagRFP (injection control) (Fig 8A). The EGFP and TagRFP mRNAs express constitutively, whereas EGFP-3x-miR-34a mRNA expression is strongly inhibited by miR-34a mimic, a synthetic RNA duplex containing miR-34a (Fig 8B). We first injected EGFP and TagRFP mRNAs with or without miR-34a mimic and did not see any effect of the miR-34a mimic on the EGFP signal. By contrast, imaging embryos at 16 hpf injected with EGFP-3x-miR-34a_site and TagRFP mRNAs with the negative control miRNA mimic resulted in EGFP signal stronger than that of TagRFP, but with the miR-34a mimic, the signal of EGFP relative to TagRFP dropped dramatically (Fig 8C). Due to injection variability, we analyzed images from 16 hpf and 28 hpf samples to determine how miR-34a mimic injection affects the ratio of EGFP to TagRFP. This analysis showed that miR-34a mimic reduced EGFP/TagRFP mean ratio by 79% at 16 hpf and by 80% at 28 hpf (Fig 8D). Thus, we confirm that the miR-34a mimic is effective at repressing translation and/or degrading its target mRNAs.

We then assessed how miR-34a overexpression mimic impacts apoptosis under control and DNA damage conditions. We did not identify any significant morphological phenotypes following reporter miR-34a mimic injections. Likewise, apoptosis imaging in initial experiments at 16 hpf and 28 hpf did not show elevated apoptosis due to miR-34a overexpression. We then performed an experiment where fish eggs were injected with 10μM negative control mimic or miR-34a mimic (Fig 8E). These fish were then divided into 3 groups: control treatment with 0.005% DMSO for 6 hours (24 to 30 hpf), 8 Gy gamma-irradiation (IR) at 24 hpf followed by 6-hour incubation or 100nM CPT treatment for 6 hours (24 to 30 hpf) (Fig 8E). Apoptosis imaging after these treatments showed that DNA damage induced high levels of apoptosis in CPT and IR groups compared to the DMSO group (Fig 8F). miR-34a mimic-injected zebrafish embryos treated with CPT exhibited dramatic morphological abnormalities (“High apoptosis curved”) compared to any other groups despite having comparable or even lower levels of apoptosis after 6 hours of treatment (Fig 8F). Indeed, the plot and the statistical test of phenotype categories (“Low apoptosis”, “High apoptosis normal”, “High apoptosis curved”) identified a significant difference between the control-injected and miR-34a mimic-injected groups only for the CPT treatment (Fig 8G). We further assessed cell death in control and miR-34a mimic-injected wild-type embryos after 2 and 3-hour treatment with 100 nM CPT (S9 Fig). The miR-34a mimic-injected exhibited earlier onset of both morphological changes (S9A Fig) and higher apoptosis levels (S9B Fig) consistent with their functional connection.

To better evaluate the contribution of miR-34a to hematopoietic stem cell development, we injected control and miR-34a mimics into wild-type and *miR-34a-/-* mutant oocytes and raised embryos to 3 days post-fertilization (dpf), which resulted in about 50-fold mature miR-34a over-expression (S8A Fig). However, miR-34a over-expression did not significantly suppress the elevated levels of myb-positive HSCs in these embryos (S8B and S8C Fig). These results together with those in Figs 7 and S7 show that miR-34a may control erythrocyte and stem cell production during the definitive wave of zebrafish hematopoiesis, but alternative expression methods will be required to demonstrate the rescue of the normal levels of erythrocytes and HSCs.

### miR-34a overexpression rescues up-regulation of some genes at 8 hpf but does not impact levels or induction of p53 target genes

We next aimed to demonstrate that miR-34a over-expression can suppress expression of genes which were up-regulated in *miR-34a-/-* mutants. We initially selected 12 of such up-regulated genes and for 9 of them the qPCR assays were robust. These genes belong to the PPAR signaling pathway (*fabp7b*, *adipoqa*), glycosylphosphatidylinositol (GPI)-anchor biosynthesis (*pigp*), starch and sucrose metabolism (*g6pca*.*1*, *g6pca*.*2*), lipid metabolism (*acot15*), leukocyte migration (*mmp9*), spermatogenesis/P granule organization (*tdrd7a*) or were otherwise one of the top up-regulated DEGs (*def6b*). Wild-type and *miR-34a-/-* mutant oocytes were injected with control and miR-34a mimics in the same way as described above and grown to 8 hpf, when RNA was extracted from all experimental groups. The injected miR-34a mimic activity was monitored by visualization of the co-injected reporter. Subsequent qPCR analysis confirmed that all of these genes are indeed up-regulated in *miR-34a-/-* mutants (S10 Fig). Even more strikingly, for 7 out of 9 of them we found convincing evidence that their up-regulation was at least partially suppressed by miR-34a overexpression. Some of these genes may be the direct targets of miR-34a, while others may be regulated indirectly.

To assess how miR-34a overexpression affects p53 activity and levels, we used a mature miR-34a assay consisting of a stem-loop cDNA synthesis method and the corresponding qPCR assay. Injection of miR-34a mimic results in a ~180-fold increase in mature miR-34a levels at 28 hpf compared to the negative control mimic, but this overexpression does not affect levels of p53 target genes under control conditions (S11A Fig). This argues against the direct regulation of p53 mRNA levels or translation by miR-34a. We then tested the effect of miR-34a overexpression on p53 target induction under genotoxic conditions after negative control mimic or miR-34a mimic injections and treating the resulting embryos at 24 hpf for 4 hours with 200nM CPT or 0.005% DMSO. In the wild-type control-injected embryos, CPT treatment increased mature miR-34a levels by 2.55-fold in control mimic samples, whereas after miR-34a mimic injection they increased by 177 to 330-fold (S11B Fig). However, miR-34a overexpression did not affect expression of six p53 target genes (*p53*, *mdm2*, *p21*, *puma*, *cycG1* and *miR-34a* transcript) or their induction by CPT treatment (S11C Fig). This result suggests that the observed DNA damage sensitization is likely due to direct miR-34a-mediated effects rather than the impact of miR-34a overexpression on the p53 pathway.

## Discussion

In this study, we focused on the *miR-34a* gene due to its coding for the major miR-34 species and its implications as a p53 modifier gene in Li-Fraumeni syndrome. *miR-34a* loss was not compensated by miR-34b and miR-34c expression. As in previous studies [22,23], we did not observe significant differences in DNA damage-induced apoptosis and p53 target expression induction between wild-type and *miR-34a-/-* mutant zebrafish embryos at 28 hpf; DNA damage also resulted in comparable DEGs in both zebrafish strains, with relatively few genes affected by lack of miR-34a.

### *miR-34* genes as *bona fide* p53 targets in zebrafish and miR-34a overexpression

We determined that in zebrafish, both *miR-34a* and *miR-34b/c* genes are primarily dependent on p53 for induction under DNA damage conditions and partially dependent on p53 for their baseline expression. Some residual induction of *miR-34* genes could be due to other transcription factors activated by DNA damage. We also predicted highly significant p53 motifs closely located to both *miR-34* genes and in similar configurations, but the formal confirmation of these p53 binding sites will require future studies. These results are fully consistent with multiple studies in mouse and human cell lines that first implicated *miR-34* genes in the p53 regulatory network [9–12]. A subsequent *in vivo* study demonstrated that in adult p53-null mice, miR-34a levels were reduced in some tissues but in major expression sites remained the same or higher [23]. Adult tissue analysis of miR-34 molecules in p53-null zebrafish may provide additional information on the dependence of *miR-34* genes on p53.

Overexpression of miR-34a has been one of the earliest tools to understand its function. Under genotoxic conditions, we indeed observed induction by p53 of *miR-34a* up to about 15-fold and up to 5-fold for *miR-34b/c*. To overexpress miR-34a, we used a commercial miR-34a mimic and a fluorescent reporter system to verify a reference target gene knock-down and measured mature miR-34a levels. Although the extent of miR-34a overexpression was well above the physiological range (100 to 200-fold on average), this experiment showed that miR-34a does not significantly influence p53 target genes in zebrafish. Consistent with this finding, it was previously reported that overexpression of all miR-34 RNAs in multiple cell lines either did not affect or upregulate p53 activity [22]. Embryos overexpressing miR-34a were much more sensitive to topoisomerase inhibition (CPT treatment) in terms of morphological defects and earlier kinetics of apoptosis induction than control-injected embryos, whereas after gamma irradiation (IR), both types of embryos responded similarly. miR-34a mimic-injected embryos may experience a higher cumulative amount of cell death over the course of continuous DNA damage due to CPT treatment than after a rapid short burst of irradiation, after which the embryo can repair some of the DNA damage. This proposed higher CPT-induced level of apoptosis in miR-34a mimic-injected embryos can be best explained by their sensitization to prolonged DNA damage. This result is consistent with previous miR-34a overexpression studies which reported increased mitotic catastrophe rates and aberrant DNA repair [61], as well as sensitization of lung cancer cells and tumors toward irradiation via inhibition of RAD51 [62].

### miR-34a loss accelerates tumor burden highlighting its tumor suppressive contributions

We established positive contributions of miR-34a to tumor suppression by comparing two single *tp53* mutants with their compound (*miR-34a-/-*) counterparts and observed earlier tumor incidence. This increased tumor burden is not simply additive since we did not observe any tumors develop in *miR-34a-/-* adult zebrafish. One caveat to these results is that *tp53-/-* fish are in a different background strain (CG1 [33]) than the *miR-34a-/-* zebrafish (*casper* [46]), which does not allow for the elimination of background-dependent effects on tumor susceptibility. However, such effects are unlikely a major influence since *tp53*^*R217H/ R217H*^ and *miR-34a-/-* both originate from the *casper* strain. The more pronounced cancer incidence differences between *tp53*^*R217H/ R217H*^ and *miR-34a-/-*; *tp53^R217H/ R217H^* fish could likely stem from the notion that miR-34a represses negative regulators of mammalian *TP53* such as MDM4 [17,20], SIRT1 [21] and HDAC1 [63]. Thus, if similar regulatory systems exist in zebrafish, the lack of miR-34a may decrease the level or activity of p53 making its level closer to the p53-null situation. In embryonic samples, we did not obtain evidence to support such regulation of wild-type *tp53* by miR-34a, but we can’t exclude it at other stages and in missense p53 mutants. These results are supported by the findings that Kras^G12D^-induced lung adenocarcinomas are promoted by both p53 haploinsufficiency and lack of miR-34a in the mouse [17]. Double inactivation of p53 and miR-34a in intestinal epithelial cells exacerbated cancer burden in a chemically-induced colorectal cancer mouse model compared to any other genotype [16], a finding fully consistent with the results reported here. Another study of Kras-mediated lung oncogenesis showed that the triple miR-34 knock-out dramatically increased the rate of cancer development and that *miR-34a* and *miR-34b/c* genes had differential effects in multiple assays [64]. The study of intestinal tumorigenesis due to an *APC*^*Min*^ mutation in mice showed that inactivation of all three *miR-34* genes was required to observe significantly more rapid mortalities, as well as dramatically altered tumor properties [18]. However, the roles played by *miR-34* genes in tumor suppression *in vivo* are highly context-dependent and subtle. The triple knock-out miR-34 study in mice showed no increased tumor incidence in these animals under normal conditions nor upon *Myc* oncogene expression, but the authors contended that in other tumor types the situation could be different based on higher *miR-34* gene levels in the corresponding tissues [23]. A recent study questioned the tumor suppressor function of *miR*-34a due to evidence that *miR-34a* is not preferentially repressed or mutated in cancer samples; its loss does not provide a proliferative advantage and only extreme overexpression can block proliferation [65]. In summary, *miR-34* genes likely have some tumor-suppressive properties but may not qualify as classically defined tumor suppressors. Indeed, our findings of more rapid tumor development upon miR-34a loss in *tp53* mutants argue for its role in tumour suppression. These tumor-suppressive contributions should be investigated further to validate the findings and their potential translational application.

### miR-34a has a role in metabolic regulation and apoptosis-related cell signaling

Identifying expression changes after a microRNA expression perturbation helps define its functions. We expression-profiled wild-type and miR-34a deficient samples at 8, 28 and 72 hpf developmental stages and identified varying numbers of DEGs, but no statistically significant de-repression of predicted miR-34 targets was observed. Since we maintained *miR-34a-/-* mutants as an independent homozygous line to prevent maternal contributions of miR-34a, our wild-type samples are not from sibling embryos and can potentially be somewhat genetically divergent. This genetic divergence can contribute to variability in gene expression. Pathway and gene ontology terms enriched among gene sets at more than one stage are likely to be more significant and relevant. Carbohydrate metabolism terms were present among gene sets at all 3 stages, but not the same genes nor direction of regulation. Although *pck1*, a key gluconeogenesis enzyme gene, was down-regulated in *miR-34a* mutant embryos at two stages, other gluconeogenesis genes were not coordinately regulated. These effects of miR-34a could be stage- and tissue-specific since at 3 dpf gluconeogenesis genes are most strongly expressed in the liver [66]. Thus, a general role of miR-34a as a gluconeogenesis regulator is still uncertain, and the importance of miR-34a regulation of gluconeogenesis genes for tumor suppression should be examined in future metabolomic experiments in the adult zebrafish. Down-regulation of *egln3 (phd3)*, a known gene involved in hypoxia regulation, may also be linked to down-regulation of gluconeogenesis genes *g6pca*.*1*, *g6pca*.*2* and *pck1* at 28 hpf, since similar findings were obtained in *Phd1-3* knockout mice and primary hepatocytes upon *Phd3* knock-down [67,68]. Another common theme is the large number of metabolic terms and pathways identified at 8 and 72 hpf, but the commonalities were limited. Apoptosis-related terms were also enriched at 8 and 72 hpf, but most of the associated genes were involved in cell signaling related to apoptosis rather than its effector functions.

### miR-34a has a role in red blood cell development at the HSC level

Given the observed up-regulation of erythrocyte markers in *miR-34a* mutant zebrafish larvae and an actual erythrocyte expansion in concert with the likely underlying increase in *myb*-positive stem cell numbers, we believe that miR-34a negatively regulates levels of hematopoietic stem cells. The HSC marker (*runx1*, *myb*) transcript levels were not globally increased in *miR-34a* deficient mutants, but the focused analysis of *myb* staining in the hematopoietic tissues showed an increase in its HSC labeling intensity at the 3 dpf larval stage. Although we were not able to suppress increased *myb* labeling with artificial miR-34a over-expression likely due to miRNA mimic limitations, we believe that these hematopoiesis-related findings are very significant and future transgenic/knock-in methods will help verify them. Since miR-34a targets MYB expression in human erythroleukemia cells [69], this result indicates MYB as a conserved miR-34a target. miR-34a is not known to regulate hematopoietic stem cells in animal models, but our results are consistent with the general inhibitory role of miR-34a expression on a “stemness” phenotype [19]. Generation of mouse induced pluripotent stem cells (reprogramming) was enhanced upon genetic inactivation of *MiR-34a* or *MiR-34b/c* [70]. Multiple studies have also documented inhibitory effects of miR-34s upon many types of cancer stem cells [71,72], but for this study, more relevant comparisons would be animal model studies of non-cancer stem cells. For example, intestinal stem cell numbers were increased in the triple *miR-34* knock-out heterozygous for an *Apc* mutation [18], which is consistent with our results in zebrafish larval HSCs. However, it is possible that miR-34a-mediated regulation of HSCs is limited to certain species such as zebrafish or earlier blood developmental stages. Thus, our results may serve as a starting point for evaluating the detailed roles of miR-34 species in HSCs and other kinds of stem cells in other animal species.

Taken together, our results show multiple nuanced but important roles for miR-34a in p53-mediated responses to DNA damage and tumour suppression as well as independent roles in metabolic regulation and blood development. This study opens new avenues for uncovering additional *miR-34a* and associated gene family functions in the zebrafish and for corroborating the conservation and *in vivo* relevance of these novel findings in other model systems.

## Supporting information

S1 Table Oligonucleotides and primers used in the study. (DOCX)

S2 Table Predicted p53 binding sites near the miR-34 genes in zebrafish. (DOCX)

S1 Fig Generation of a *miR-34a* deletion mutant and its verification. **(A)** A diagram of the genomic region containing the *miR-34a* gene and sgRNAs used for generating the deletion. **(B)** Initial genotyping of embryos injected with 6 sgRNAs and Cas9 RNA. The bands from the *miR-34a* PCR assay that are not present in the wild-type samples are deletion products. **(C)** F0 founder screening by genotyping pools of their F1 progeny. F0 fish were bred to wild-type fish to produce the F1 progeny embryos, 30 of which were genotyped with the same assay as in **(B)**. The lower bands are PCR products from the deleted alleles of *miR-34a*. **(D)** Sequencing of the PCR products resulting from *miR-34a* deletion. **(E)** qPCR analysis of *miR-34* genes (a,b,c) in 28 hpf embryo RNA samples from wild-type and *miR-34a-/-* (miR-34a-del) fish (n = 4 of pooled embryo samples). The low value *miR-34a* gene in its deletion mutant represents qPCR background.
(DOCX)

S2 Fig Normal apoptosis induction by DNA damage in *miR-34a*-/- mutant zebrafish embryos. Wild-type and *miR-34a*-/- embryos (groups of 30 each) at ~22 hpf were incubated for 6 hours with 0.005% DMSO, 0.25, 0.5 or 1 μM Camptothecin. Representative Acridine Orange (AO) stained embryos are shown at the experimental endpoint at ~28 hpf.
(DOCX)

S3 Fig Clustering of normalized read counts of known p53 target genes induced or repressed by DNA damage due to camptothecin treatment. Heatmap for hierarchical clustering of gene expression values for known p53 target genes. Both up- and down-regulated p53 target genes are shown in the heatmap. Values for different groups have been averaged to simplify presentation. Treatment and genotype assignments are indicated by color bars above the heatmap and the legend is provided on the side.
(DOCX)

S4 Fig Comparison of R217H *tp53* transcript levels in *tp53^R217/R217H^* larval and adult tissues to the tp53 expression in wild-type 6 dpf larvae. **(A)** Genomic *tp53* amplicon-based genotyping of 6 dpf wild-type and *miR-34a-/-;tp53*^*R217H/R217H*^ as well as of *tp53*^*R217H/R217H*^ and *miR-34a-/-;tp53*^*R217H/R217H*^ tumor and eye samples. **(B)** Amplification of ~700-bp p53 cDNA fragment from the cDNA derived from the RNAs analyzed in (A). **(C)** Quantitative PCR to measure the expression of *tp53* transcript in all of the samples from (B) relative to the 6 dpf wild-type larvae.
(DOCX)

S5 Fig Gene Ontology REVIGO graphs for 8 and 72 hpf RNA-Seq datasets. (DOCX)

S6 Fig Gluconeogenesis gene expression in RNA-Seq datasets. Bar graph of normalized relative expression values of gluconeogenesis-related genes (*pck1*, *pck2*, *fam3a*, *g6pca*.*1*, *g6pca*.*2*) in the 8, 28 and 72 hpf RNA-seq datasets comprising data on wild-type and *miR-34a*^-/-^ RNA samples. The y-scale is in the log10 scale. The significance of differences was derived from the RNA-seq false-discovery rate values and are indicated by ‘***’ (P-value < 0.001), ‘**’ (P-value < 0.01) and ‘*’ (P-value < 0.05).
(DOCX)

S7 Fig *miR-34a-/-* mutant embryos at 3 dpf have elevated expression levels of *alas2* erythrocyte marker and *myb* hematopoietic stem cell marker relative to the wild-type embryos. (**A**) Representative *alas2 in situ* staining images of 3 dpf wild-type and *miR-34a-/-* embryos of ‘High’ and ‘Low’ phenotype categories. The numbers of embryos are indicated. The staining was performed on embryos from three independent samples. (**B**) A proportional stacked bar graph of ‘High’ and ‘Low’ phenotype categories of of both genotypes. The significance of both Fisher’s exact test (P-value = 0.005937) and the Chi-square test (P-value = 0.005193) is indicated above the graph with “**”. (**C**) Representative *myb in situ* staining images of the caudal hematopoietic tissue regions of 3 dpf wild-type and *miR-34a-/-* embryos. The numbers of embryos are indicated. The staining was performed on embryos from three independent samples. (**D**) Quantification of *myb* staining using the Ilastik-Cell Profiler pixel classification approach. Relative values of positively classified pixels (fold change) are shown. The numbers of embryos are the same as in (**C**). The significance of the differences between the genotypes was calculated by the two-sample t-test (***; P-value < 2.2e-16).
(DOCX)

S8 Fig miR-34a mimic does not suppress *myb* expression up-regulation in *miR-34a-/-* mutants. (**A**) Mature miR-34a qPCR analysis in 3 dpf wild-type and *miR-34a-/-* embryos injected with control or miR-34a mimics (n = 4 of pooled RNA samples for each condition). (**B**) Quantification of *myb* staining using the Ilastik-Cell Profiler pixel classification approach. Relative areas of positively classified pixels (fold change) are shown. The numbers of embryos are the same as in (**C**). The significances of the differences in (**A**) and (**B**) were determined by one−way ANOVA with a Tukey’s post−hoc test (***—P-value < 0.001), error bars represent standard errors of the mean, each point represents a pooled RNA sample (A) or an individual stained embryo (**B**). (**C**) Representative *myb in situ* staining images of the caudal hematopoietic tissue regions of 3 dpf wild-type and *miR-34a-/-* embryos injected with control or miR-34a mimics. The numbers of embryos are indicated. The staining was performed on embryos from two independent experiments.
(DOCX)

S9 Fig miR-34a over-expression by mimic injection sensitizes zebrafish to earlier apoptosis induction. (**A**) Imaging of TUNEL and DAPI staining of wild-type embryos injected with control or miR-34a mimics and treated with DMSO for 2 hours or 100 nM camptothecin (CPT) for 2 or 3 hours. Developmental stages, treatments and total numbers of imaged and quantified embryos are shown on a representative embryos image. (**B**) A plot of the relative apoptotic index (TUNEL staining area determined using the Ilastik-Cell Profiler and divided by the embryo-body (without the yolk) DAPI labeling area) for all treatment groups. ANOVA analysis of the injection and treatment factors was done, which is indicated by grouping both CPT-treated samples for both injection types. The significances of the differences between groups were determined by a Tukey’s post−hoc test (**—P-value < 0.01; *—P-value < 0.05), error bars represent standard errors of the mean, each point represents an individual stained embryo.
(DOCX)

S10 Fig miR-34a over-expression decreases expression of some genes up-regulated in *miR-34a-/-* mutants at 8 hpf. qPCR analysis of 9 genes in 8 hpf wild-type and *miR-34a-/-* embryos injected with control or miR-34a mimics (n = 6 of pooled RNA samples for each condition). Fold changes relative to the wildtype injected with control mimics are indicated on the y-axis and experimental groups–on the x-axis. ANOVA analysis of the experimental groups vs Cq values was done for each gene. The significances of the differences between groups were determined by a Tukey’s post−hoc test (***—P-value < 0.001; **—P-value < 0.01; *—P-value < 0.05), error bars represent standard errors of the mean, each point represents a pooled RNA sample. Some of the less relevant differences were omitted. The data is from two independent experiments.
(DOCX)

S11 Fig No effect of miR-34a mimic over-expression on p53 target expression at 28 hpf. (**A**) Mature miR-34a (*miR34a*), *p53*, *p21*, *puma* and *mdm2* mRNA levels were analyzed at 28 hpf in control mimic-injected or in miR-34a mimic-injected embryos. The resulting relative expression levels (Fold change) are plotted in the log10 scale. n = 4 for each type of sample. Significance was determined by a t-test and indicated by “***”–P-value < 0.001. (**B**) and (**C**) are parts of one experiment repeated twice with 3 biological replicates each aimed at testing if miR-34a over-expression influences p53 target induction by 4-hour treatment with 200 nM camptothecin (CPT). ANOVA was used to measure differences (“***”–P-value < 0.001). (**B**) Mature miR-34a levels in control mimic-injected or in miR-34a mimic-injected 28 hpf zebrafish embryos treated with 0.005% DMSO or with 200 nM CPT for 4 hours. Levels of mature miR-34a in miR-34a mimic samples are artificial due to injection and “***” indicates their difference from the wild-type samples, but not from each other. (**C**) p53 target (*p53*, *mdm2*, *p21*, *puma*, *cycG1* and *miR34a*) expression in control mimic-injected or in miR-34a mimic-injected 28 hpf zebrafish embryos treated with 0.005% DMSO or with 200 nM CPT for 4 hours. Significant induction of the genes is indicated by “***” and signifies the difference in their expression between respective CPT and control samples. None of the other differences were detected.
(DOCX)

S1 File –Tables of DEGs due to treatment by camptothecin treatment in wild-type, miR-34a mutant and both genotype zebrafish embryos at 28 hpf. (XLSX)

S2 File –Table of DEGs due to *miR-34a* loss under control conditions at 28 hpf. (XLSX)

S3 File –Tables of GO BP and KEGG Pathway terms enriched among the DEGs due to *miR-34a* loss under control conditions at 28 hpf. (XLSX)

S4 File –Zebrafish orthologues of known p53 targets and TargetScan predicted miR-34 targets). (XLSX)

S5 File –Tables of DEGs due to *miR-34a* loss at 8 and 72 hpf and GO BP and KEGG Pathway terms enriched in these datasets. (XLSX)

S6 File –ZEOGS analysis results of up- and down-regulated genes from the 72 hpf dataset. (XLSX)
