# Supplementary material for: miR-34a is a tumor suppressor in zebrafish and its expression levels impact metabolism, hematopoiesis and DNA damage
Source: PLoS Genet. 2024 May 28;20(5):e1011290. doi: 10.1371/journal.pgen.1011290 (PMC11166285; doi:10.1371/journal.pgen.1011290)
Supplement: S1 Table — (DOCX) [file pgen.1011290.s001.docx]

**S1 Table. Oligonucleotides and primers used in the study.**

| **Name** | **Sequence** |
| --- | --- |
| sgRNA-miR34a-1 | GTAATACGACTCACTATAGGAGATGCTGAAGCGAGCGAGTTTTAGAGCTAGAAATAGC |
| sgRNA-miR34a-3 | GTAATACGACTCACTATAGGCAGGCTCTGAATGCTCGAGCGTTTTAGAGCTAGAAATAGC |
| sgRNA-miR34a-375_sense | GTAATACGACTCACTATAGGCGGACGTTTAATGAGCAGGTTTTAGAGCTAGAAATAGC |
| sgRNA-miR34a-427_sense | GTAATACGACTCACTATAGGCGGGACAGTCGTTAAAACAGTTTTAGAGCTAGAAATAGC |
| sgRNA-miR34a-629_sense | GTAATACGACTCACTATAGGCTCTGAATGCTCGAGCGGTTTTAGAGCTAGAAATAGC |
| sgRNA-miR34a-704_sense | GTAATACGACTCACTATAGGATTAGAAGAATGAATGAGAGTTTTAGAGCTAGAAATAGC |
| rev_sgRNA_scaffold | AGCACCGACTCGGTGCCACTTTTTCAAGTTGATAACGGACTAGCCTTATTTTAACTTGCTATTTCTAGCTCTAAAAC |
| miR-34a_assay_for | TACCTCTGTCCCTGGCTGTC |
| miR-34a_assay_rev | TGAATCACTCCTGCTTTCATTG |
| R217_SA_for | AAATTGCCAGAGTATGTGTCTGTCC |
| R217_SA_rev | ATGAGAGCAGCATCATGAAGCAT |
| p53_null_for | CGCAAGGTCAACAAGTGCTA |
| p53_null_rev | TTGCCTTGTTCTCTGCAGTTT |
| p53cDNA_for | ATGGCGCAAAACGACAGCCA |
| p53cDNA_rev | AGACCTCCGGCCCAGCAACT |
| miR34a-3xPT_rep_for | TCGAgaatctagaACAACCAGCTAAGACACTGCCAtagtaACAACCAGCTAAGACACTGCCAtagtaACAACCAGCTAAGACACTGCCAg |
| miR34a-3xPT_rep_rev | CTAGcTGGCAGTGTCTTAGCTGGTTGTtactaTGGCAGTGTCTTAGCTGGTTGTtactaTGGCAGTGTCTTAGCTGGTTGTtctagattc |
| mir-34a_ISH_for | CGCCTCACCTATACCGTCAT |
| T7-mir-34a_ISH_rev | GTAATACGACTCACTATAGGGAATGAGACGGGAAGCTTGA |
| 18s_rrna_qfor | CGGAAAGGATTGACAGATTGATA |
| 18s_rrna_qrev | CGCTCCACCAACTAAGAACG |
| eef1a1a_qfor | CCAGCAAATACTACGTCACCAT |
| eef1a1a_qrev | CAATCAGCACAGCACAATCC |
| cycG1-qfor | CATCTCTAAAAGAGGCTCTAGATGG |
| cycG1-qrev | CACACAAACCAGGTCTCCAG |
| p21_qfor | AGCTGCATTCGTCTCGTAGC |
| p21_qrev | TGAGAACTTACTGGCAGCTTCA |
| mir34a_qfor | CTGCTGTGAGTGGTTCTCTGG |
| mir34a_qrev | GCGGCAGTATACTTGCTGATT |
| miR34b_qfor | GGGTTGGTCTGTAGGCAGTG |
| miR34b_qrev | TTGTTGTGTTGGCAGTATGG |
| mir34c_qfor | TGTGTGGTCACCAGGCAGT |
| mir34c_qrev | TAGCCTTCACCTGGTAGTGAGG |
| p53q_for | CCCATCCTCACAATCATCACT |
| p53q_rev | CACGCACCTCAAAAGACCTC |
| mdm2_qfor | GATGCAGGTGCAGATAAAGATG |
| mdm2_qrev | CCTTGCTCATGATATATTTCCCTAA |
| puma_qfor | GAACACACGGGTTACAAAGGAC |
| puma_qrev | GAAAAATCCCAGAGTCTGTAAGTG |
| miR-34a_SL_RT | GAAAGAAGGCGAGGAGCAGATCGAGGAAGAAGACGGAAGAATGTGCGTCTCGCCTTCTTTCacaaccag |
| U6_RT_primer | AAAAATATGGAGCGCTTCACG |
| miR-34a_mature_o2_for | GGTGGCAGTGTCTTAGCT |
| Rev_SL_qPCR_rev | CGAGGAAGAAGACGGAAGAAT |
| U6_for | TTGGTCTGATCTGGCACATATAC |
| U6_rev | AAAAATATGGAGCGCTTCACG |
| hbae1_qfor | CCAGGATGTTGATTGTCTAC |
| hbae1_qrev | CAGTCTTGCCGTGTTTC |
| hbae3_qfor | CCTAAGCCCCAACTCTC |
| hbae3_qrev | CTCCCTTCAGGTCATCC |
| hbbe1_qfor | CTTGACCATCGTTGTTG |
| hbbe1_qrev | GATGAATTTCTGGAAAGC |
| hbbe2_qfor | GTGCTGCACTCCGAGAAACT |
| hbbe2_qrev | TCACGATGGTCAGGCAGTC |
| hbae1_qfor | CCAGGATGTTGATTGTCTAC |
| hbae1_qrev | CAGTCTTGCCGTGTTTC |
| hbae3_qfor | CCTAAGCCCCAACTCTC |
| lcp1_qfor | GGTCAACAAACCACCATATCC |
| lcp1_qrev | GCTCCACCGCATAGTTACAGT |
| cpa5_qfor | CAGCTGCGTTGGAGTTGA |
| cpa5_qrev | TTGCTGGAACCACCACCT |
| mpx_qfor | TGCTGTTGTGCTCTTTCAATG |
| mpx_qrev | TGAATCAAGAATAAAAGGTCTTCCA |
| runx1_qfor | TTTGGGACGCCAAATACG |
| runx1_qrev | AAACCCTCGCTCATCTTCC |
| spi1a_qfor | AGCACCTCCAGCACTCTGTC |
| spi1a_qrev | CGTCCTGAAGGAGGTCCA |
| mpeg1.1_qfor | TGTTACAGCACGGGTTCAAG |
| mpeg1.1_qrev | AATGGCGTCAGCGATTTC |
| myb_qfor | CGCCAGCTTTCTACCGAAT |
| myb_qrev | CAGGGTTGAGGACTTTCTGC |
| g6pca.1_qfor | TTGTTTCAGGATTGGATGTATTG |
| g6pca.1_qrev | GAAGCAGCAAAGCCACAG |
| g6pca.2_qfor | CACACGGCTGCTCTCTTCT |
| g6pca.2_qrev | GATAAGCAGTACGGGATGATG |
| mmp9_qfor | TCATGATCTCTTTCGGGAAACT |
| mmp9_qrev | GAGCGTCTCCCTGTGTGC |
| fabp7b_qfor | AGTTTGTTTATCACAGTTTTGATTCAG |
| fabp7b_qrev | CTTGTTGCAAACCCAATACCT |
| tdrd7a_qfor | GCTCCCAGTTGCATGAAGAT |
| tdrd7a_qrev | GCCTCATTGCCACAGAAGA |
| pigp_qfor | GAGCCATCTACGGCTTTGTG |
| pigp_qrev | ATGGAGCCAAGAGTCAGGAA |
| def6b_qfor | GCAGAGTGGACCAGGACATC |
| def6b_qrev | CATCTGGACGTTCCAGTGTTT |
| acot15_qfor | TATCGGACGTGTCATCATGC |
| acot15_qrev | ATTCCTCGACATGGCTGAAC |
| adipoqa_qfor | ACGAGACGGACGTGATGG |
| adipoqa_qrev | TGCCTGGAAATCCTTCATCT |
| bactin_qfor | CGAGCTGTCTTCCCATCCA |
| bactin_qrev | TCACCAACGTAGCTGTCTTTCTG |
| ef1a-new_qfor | GAGAAGTTCGAGAAGGAAGCC |
| ef1a-new_qrev | AATGGTGATACCACGCTCAC |
| alas2_ISH_for | CTGAGCAAAATGGCCTTCTC |
| T7_alas2_ISH_rev | TAATACGACTCACTATAGGGCCCAGAGTTCCAGAGACAA |
| cmyb_for | CGAGGCGGCACAGACACAGTGTTT |
| T7_cmyb_rev | TAATACGACTCACTATAGGGAGACACACTCCTGGGGCTCTTGT |
