# Supplementary material for: miR-34a is a tumor suppressor in zebrafish and its expression levels impact metabolism, hematopoiesis and DNA damage
Source: PLoS Genet. 2024 May 28;20(5):e1011290. doi: 10.1371/journal.pgen.1011290 (PMC11166285; doi:10.1371/journal.pgen.1011290)
Supplement: S2 Table — (DOCX) [file pgen.1011290.s002.docx]

**S2 Table. Predicted p53 binding sites near the miR-34 genes in zebrafish**

| **Gene** | **MAST (MEME Suite)** | **Matrix-scan(RSAT Suite)** | **Relative coordinate** |
| --- | --- | --- | --- |
| miR-34a | CACATGTCTAAACATAAT | CACATGTCTAAACATAATA | -1813 |
|  | GACATGGCGTGACCTGTT | GACATGGCGTGACCTGTTT | +430 |
| miR-34b/c | GGCAAGTCTAGACCTGTT | GGCAAGTCTAGACCTGTTT | -1785 |
|  | AACATGTTTGATCATAAA | Not found | +1581 |
|  | GTCTTGCTCGCACAAGCT | GTCTTGCTCGCACAAGCTT | +8266 |
