# Supplementary material for: miR-34a is a tumor suppressor in zebrafish and its expression levels impact metabolism, hematopoiesis and DNA damage
Source: PLoS Genet. 2024 May 28;20(5):e1011290. doi: 10.1371/journal.pgen.1011290 (PMC11166285; doi:10.1371/journal.pgen.1011290)
Supplement: S1 Fig — (A) A diagram of the genomic region containing the miR-34a gene and sgRNAs used for generating the deletion. (B) Initial genotyping of embryos injected with 6 sgRNAs and Cas9 RNA. The bands from the miR-34a PCR assay that are not present in the wild-type samples are deletion products. (C) F0 founder screening by genotyping pools of their F1 progeny. F0 fish were bred to wild-type fish to produce the F1 progeny embryos, 30 of which were genotyped with the same assay as in (B). The lower bands are PCR products from the deleted alleles of miR-34a. (D) Sequencing of the PCR products resulting from miR-34a deletion. (E) qPCR analysis of miR-34 genes (a,b,c) in 28 hpf embryo RNA samples from wild-type and miR-34a-/- (miR-34a-del) fish (n = 4 of pooled embryo samples). The low value miR-34a gene in its deletion mutant represents qPCR background. (DOCX) [file pgen.1011290.s003.docx]

**
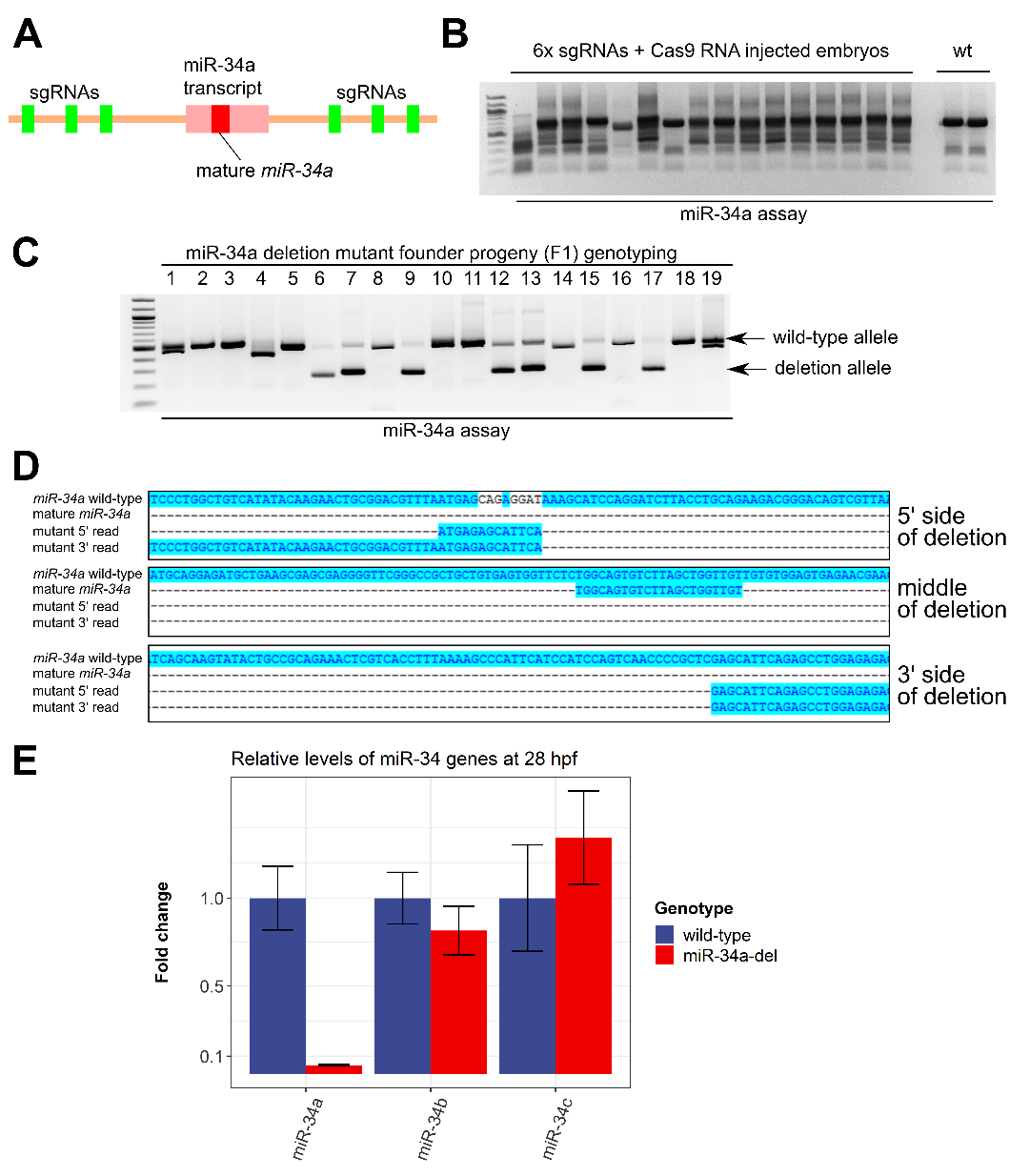
**

**Figure S1. Generation of a *miR-34a* deletion mutant and its verification.**

**(A)** A diagram of the genomic region containing the *miR-34a* gene and sgRNAs used for generating the deletion. **(B)** Initial genotyping of embryos injected with 6 sgRNAs and Cas9 RNA. The bands from the *miR-34a* PCR assay that are not present in the wild-type samples are deletion products. **(C)** F0 founder screening by genotyping pools of their F1 progeny. F0 fish were bred to wild-type fish to produce the F1 progeny embryos, 30 of which were genotyped with the same assay as in **(B)**. The lower bands are PCR products from the deleted alleles of *miR-34a*. **(D)** Sequencing of the PCR products resulting from *miR-34a* deletion. **(E)** qPCR analysis of *miR-34* genes (a,b,c) in 28 hpf embryo RNA samples from wild-type and *miR-34a-/-* (miR-34a-del) fish (n = 4 of pooled embryo samples). The low value *miR-34a* gene in its deletion mutant represents qPCR background.
