# Supplementary material for: miR-34a is a tumor suppressor in zebrafish and its expression levels impact metabolism, hematopoiesis and DNA damage
Source: PLoS Genet. 2024 May 28;20(5):e1011290. doi: 10.1371/journal.pgen.1011290 (PMC11166285; doi:10.1371/journal.pgen.1011290)
Supplement: S2 Fig — Wild-type and miR-34a-/- embryos (groups of 30 each) at ~22 hpf were incubated for 6 hours with 0.005% DMSO, 0.25, 0.5 or 1 μM Camptothecin. Representative Acridine Orange (AO) stained embryos are shown at the experimental endpoint at ~28 hpf. (DOCX) [file pgen.1011290.s004.docx]

**
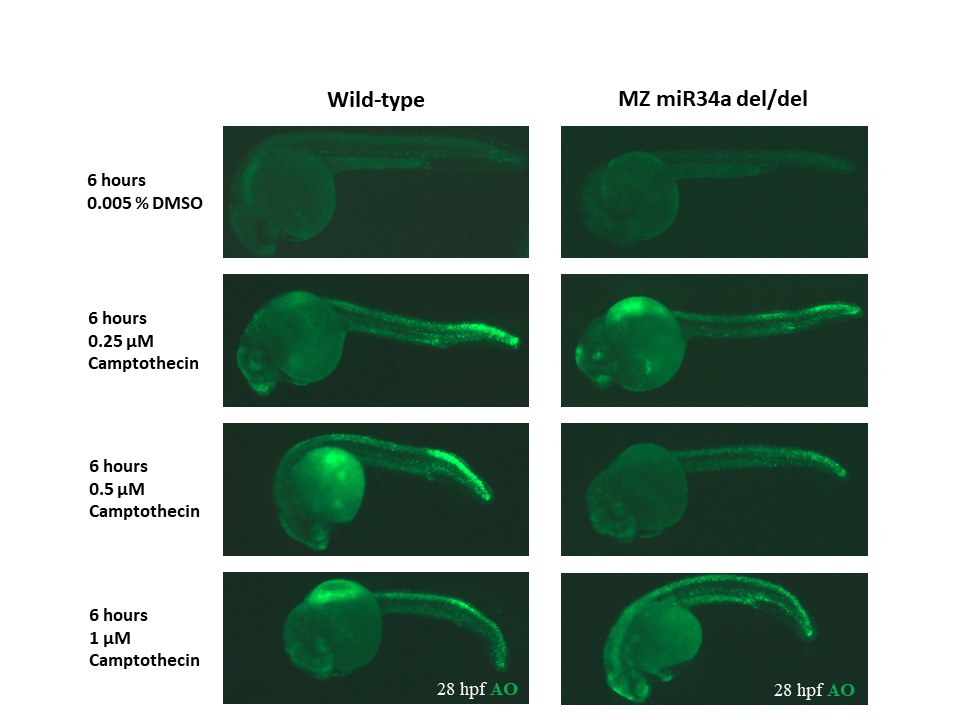
**

**Figure S2. Normal apoptosis induction by DNA damage in** ***miR-34a*-/- mutant zebrafish embryos.** Wild-type and *miR-34a*-/- embryos (groups of 30 each) at ~22 hpf were incubated for 6 hours with 0.005% DMSO, 0.25, 0.5 or 1 µM Camptothecin. Representative Acridine Orange (AO) stained embryos are shown at the experimental endpoint at ~28 hpf.
