# Supplementary material for: miR-34a is a tumor suppressor in zebrafish and its expression levels impact metabolism, hematopoiesis and DNA damage
Source: PLoS Genet. 2024 May 28;20(5):e1011290. doi: 10.1371/journal.pgen.1011290 (PMC11166285; doi:10.1371/journal.pgen.1011290)
Supplement: S3 Fig — Heatmap for hierarchical clustering of gene expression values for known p53 target genes. Both up- and down-regulated p53 target genes are shown in the heatmap. Values for different groups have been averaged to simplify presentation. Treatment and genotype assignments are indicated by color bars above the heatmap and the legend is provided on the side. (DOCX) [file pgen.1011290.s005.docx]

**
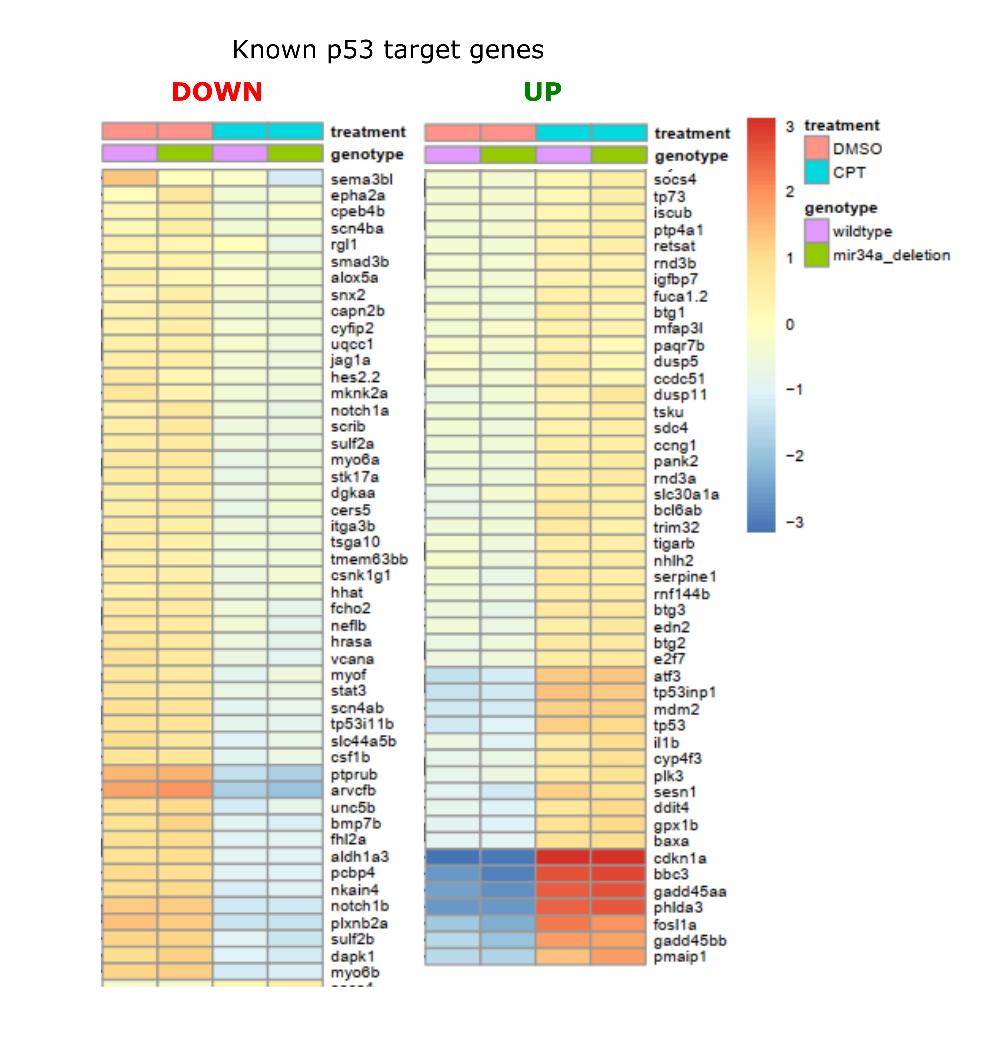
**

**Figure S3. Clustering of normalized read counts of known p53 target genes induced or repressed by DNA damage due to camptothecin treatment.** Heatmap for hierarchical clustering of gene expression values for known p53 target genes. Both up- and down-regulated p53 target genes are shown in the heatmap. Values for different groups have been averaged to simplify presentation. Treatment and genotype assignments are indicated by color bars above the heatmap and the legend is provided on the side.
