# Supplementary material for: miR-34a is a tumor suppressor in zebrafish and its expression levels impact metabolism, hematopoiesis and DNA damage
Source: PLoS Genet. 2024 May 28;20(5):e1011290. doi: 10.1371/journal.pgen.1011290 (PMC11166285; doi:10.1371/journal.pgen.1011290)
Supplement: S4 Fig — (A) Genomic tp53 amplicon-based genotyping of 6 dpf wild-type and miR-34a-/-;tp53R217H/R217H as well as of tp53R217H/R217H and miR-34a-/-;tp53R217H/R217H tumor and eye samples. (B) Amplification of ~700-bp p53 cDNA fragment from the cDNA derived from the RNAs analyzed in (A). (C) Quantitative PCR to measure the expression of tp53 transcript in all of the samples from (B) relative to the 6 dpf wild-type larvae. (DOCX) [file pgen.1011290.s006.docx]

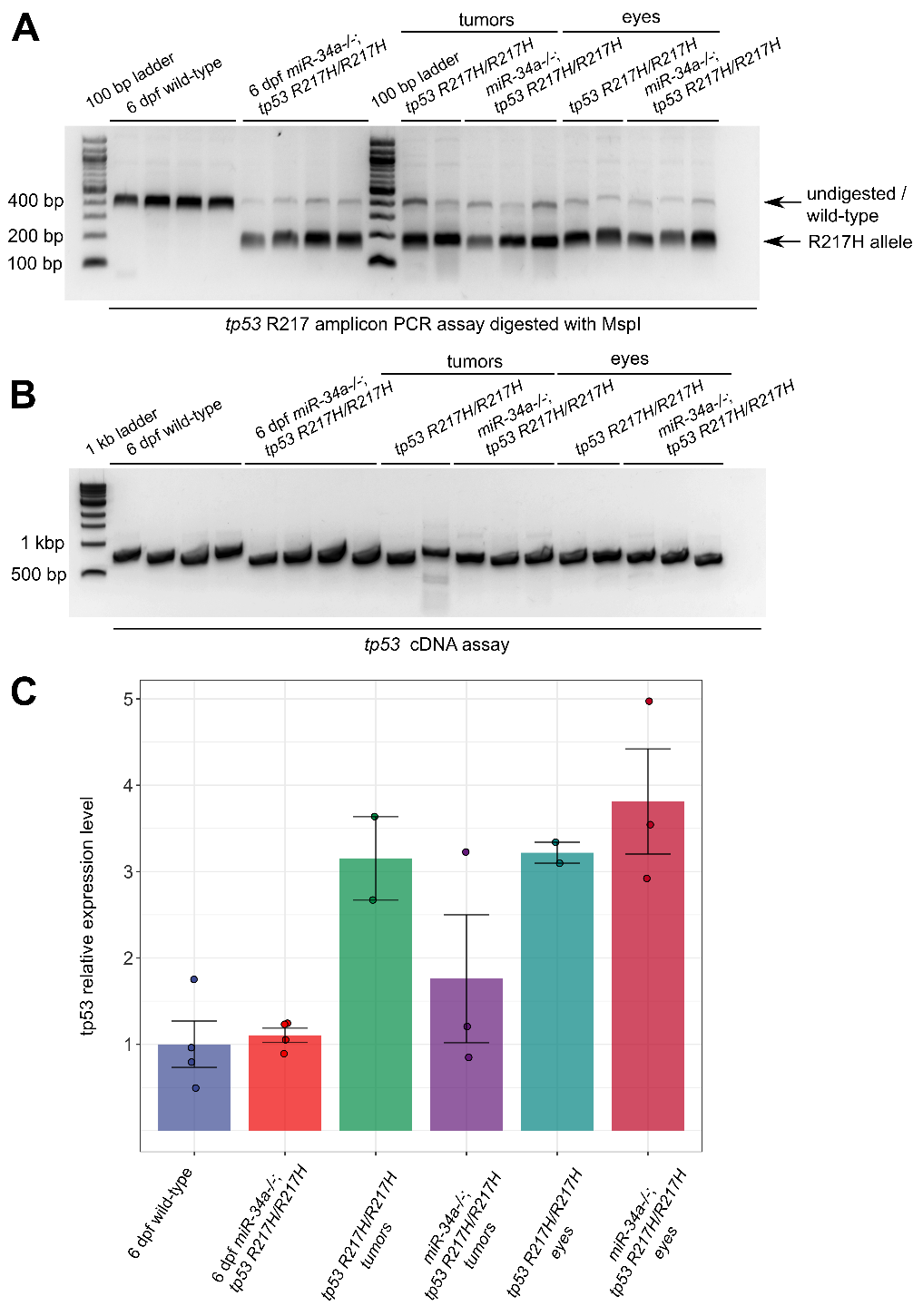


**Figure S4. Comparison of R217H tp53 transcript levels in tp53^R217/R217H^ larval and adult tissues to the tp53 expression in wild-type 6 dpf larvae.**

**(A)** Genomic *tp53* amplicon-based genotyping of 6 dpf wild-type and *miR-34a-/-;tp53^R217H/R217H^* as well as of *tp53^R217H/R217H^* and *miR-34a-/-;tp53^R217H/R217H^* tumor and eye samples. **(B)** Amplification of ~700-bp p53 cDNA fragment from the cDNA derived from the RNAs analyzed in (A). **(C)** Quantitative PCR to measure the expression of tp53 transcript in all of the samples from (B) relative to the 6 dpf wild-type larvae.
