# Supplementary figures and images for: miR-34a is a tumor suppressor in zebrafish and its expression levels impact metabolism, hematopoiesis and DNA damage
Source: PLoS Genet. 2024 May 28;20(5):e1011290. doi: 10.1371/journal.pgen.1011290 (PMC11166285; doi:10.1371/journal.pgen.1011290)

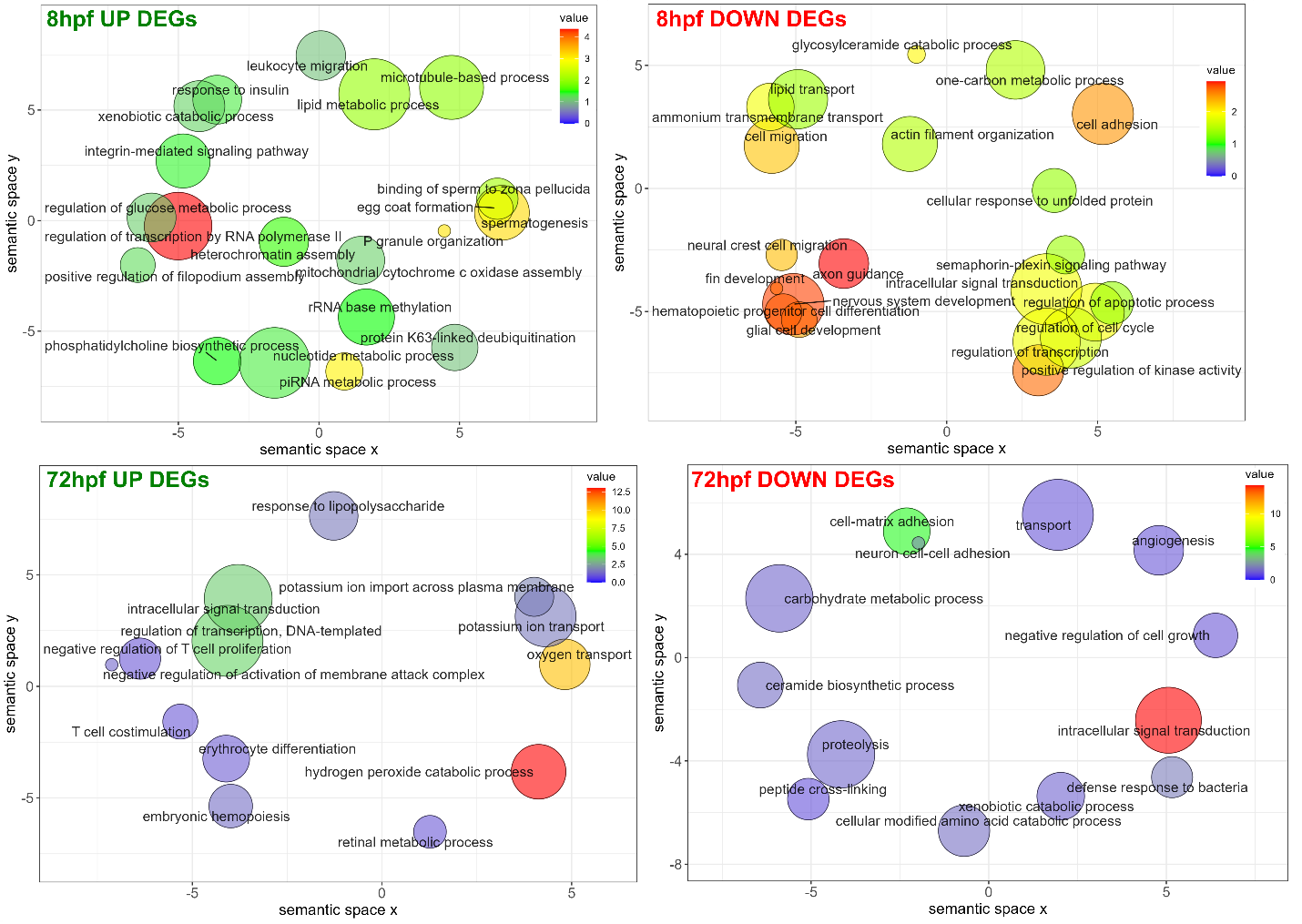


**Figure S5. Gene Ontology REVIGO graphs for 8 and 72 hpf RNA-Seq datasets.**

Supplement: S5 Fig — (DOCX) [file pgen.1011290.s007.docx]
