# Supplementary material for: miR-34a is a tumor suppressor in zebrafish and its expression levels impact metabolism, hematopoiesis and DNA damage
Source: PLoS Genet. 2024 May 28;20(5):e1011290. doi: 10.1371/journal.pgen.1011290 (PMC11166285; doi:10.1371/journal.pgen.1011290)
Supplement: S6 Fig — Bar graph of normalized relative expression values of gluconeogenesis-related genes (pck1, pck2, fam3a, g6pca.1, g6pca.2) in the 8, 28 and 72 hpf RNA-seq datasets comprising data on wild-type and miR-34a-/- RNA samples. The y-scale is in the log10 scale. The significance of differences was derived from the RNA-seq false-discovery rate values and are indicated by ‘***’ (P-value < 0.001), ‘**’ (P-value < 0.01) and ‘*’ (P-value < 0.05). (DOCX) [file pgen.1011290.s008.docx]

**
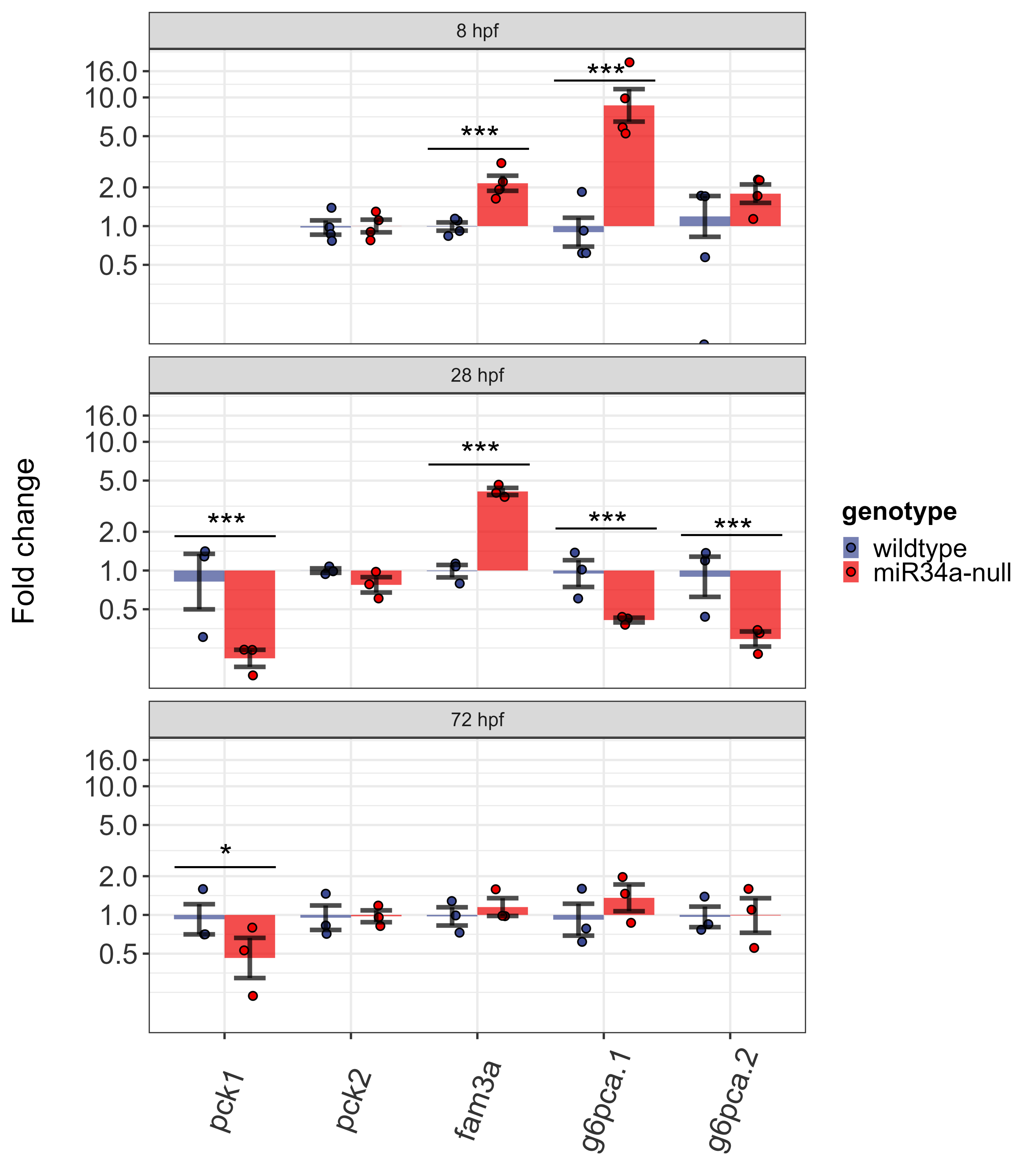
**

**Figure S6. Gluconeogenesis gene expression in RNA-Seq datasets.** Bar graph of normalized relative expression values of gluconeogenesis-related genes (*pck1, pck2, fam3a, g6pca.1, g6pca.2*) in the 8, 28 and 72 hpf RNA-seq datasets comprising data on wild-type and *miR-34a*^-/-^ RNA samples. The y-scale is in the log10 scale. The significance of differences was derived from the RNA-seq false-discovery rate values and are indicated by ‘***’ (P-value < 0.001), ‘**’ (P-value < 0.01) and ‘*’ (P-value < 0.05).
