# Supplementary material for: miR-34a is a tumor suppressor in zebrafish and its expression levels impact metabolism, hematopoiesis and DNA damage
Source: PLoS Genet. 2024 May 28;20(5):e1011290. doi: 10.1371/journal.pgen.1011290 (PMC11166285; doi:10.1371/journal.pgen.1011290)
Supplement: S7 Fig — (A) Representative alas2 in situ staining images of 3 dpf wild-type and miR-34a-/- embryos of ‘High’ and ‘Low’ phenotype categories. The numbers of embryos are indicated. The staining was performed on embryos from three independent samples. (B) A proportional stacked bar graph of ‘High’ and ‘Low’ phenotype categories of of both genotypes. The significance of both Fisher’s exact test (P-value = 0.005937) and the Chi-square test (P-value = 0.005193) is indicated above the graph with “**”. (C) Representative myb in situ staining images of the caudal hematopoietic tissue regions of 3 dpf wild-type and miR-34a-/- embryos. The numbers of embryos are indicated. The staining was performed on embryos from three independent samples. (D) Quantification of myb staining using the Ilastik-Cell Profiler pixel classification approach. Relative values of positively classified pixels (fold change) are shown. The numbers of embryos are the same as in (C). The significance of the differences between the genotypes was calculated by the two-sample t-test (***; P-value < 2.2e-16). (DOCX) [file pgen.1011290.s009.docx]

**
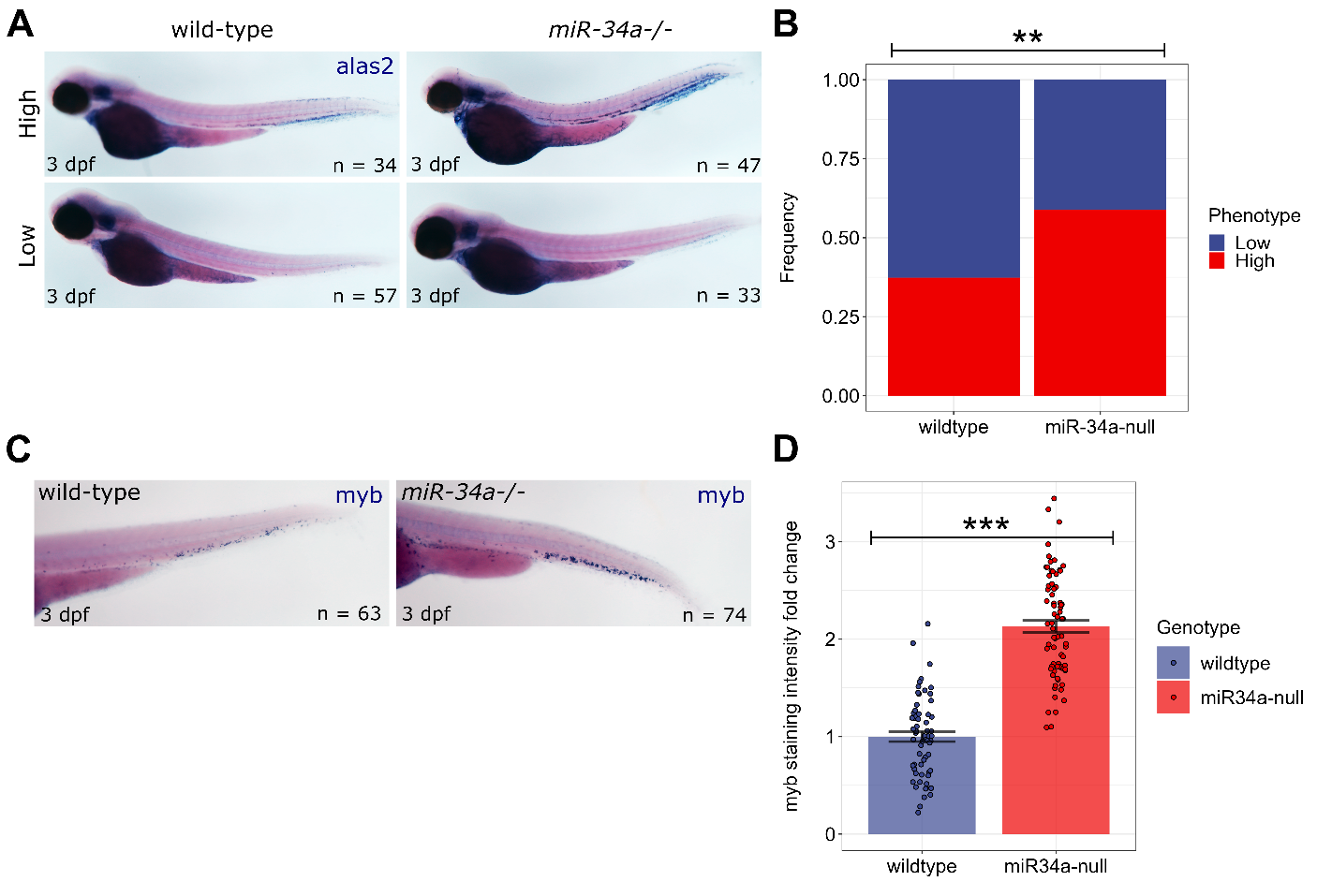
**

**Figure S7. *miR-34a-/-* mutant embryos at 3 dpf have elevated expression levels of *alas2* erythrocyte marker and *myb* hematopoietic stem cell marker relative to the wild-type embryos.**

(**A**) Representative *alas2* *in situ* staining images of 3 dpf wild-type and *miR-34a-/-* embryos of ‘High’ and ‘Low’ phenotype categories. The numbers of embryos are indicated. The staining was performed on embryos from three independent samples. (**B**) A proportional stacked bar graph of ‘High’ and ‘Low’ phenotype categories of of both genotypes. The significance of both Fisher’s exact test (P-value = 0.005937) and the Chi-square test (P-value = 0.005193) is indicated above the graph with “**”. (**C**) Representative *myb* *in situ* staining images of the caudal hematopoietic tissue regions of 3 dpf wild-type and *miR-34a-/-* embryos. The numbers of embryos are indicated. The staining was performed on embryos from three independent samples. (**D**) Quantification of *myb* staining using the Ilastik-Cell Profiler pixel classification approach. Relative values of positively classified pixels (fold change) are shown. The numbers of embryos are the same as in (**C**). The significance of the differences between the genotypes was calculated by the two-sample t-test (***; P-value < 2.2e-16).
