# Supplementary material for: miR-34a is a tumor suppressor in zebrafish and its expression levels impact metabolism, hematopoiesis and DNA damage
Source: PLoS Genet. 2024 May 28;20(5):e1011290. doi: 10.1371/journal.pgen.1011290 (PMC11166285; doi:10.1371/journal.pgen.1011290)
Supplement: S8 Fig — (A) Mature miR-34a qPCR analysis in 3 dpf wild-type and miR-34a-/- embryos injected with control or miR-34a mimics (n = 4 of pooled RNA samples for each condition). (B) Quantification of myb staining using the Ilastik-Cell Profiler pixel classification approach. Relative areas of positively classified pixels (fold change) are shown. The numbers of embryos are the same as in (C). The significances of the differences in (A) and (B) were determined by one−way ANOVA with a Tukey’s post−hoc test (***—P-value < 0.001), error bars represent standard errors of the mean, each point represents a pooled RNA sample (A) or an individual stained embryo (B). (C) Representative myb in situ staining images of the caudal hematopoietic tissue regions of 3 dpf wild-type and miR-34a-/- embryos injected with control or miR-34a mimics. The numbers of embryos are indicated. The staining was performed on embryos from two independent experiments. (DOCX) [file pgen.1011290.s010.docx]

**
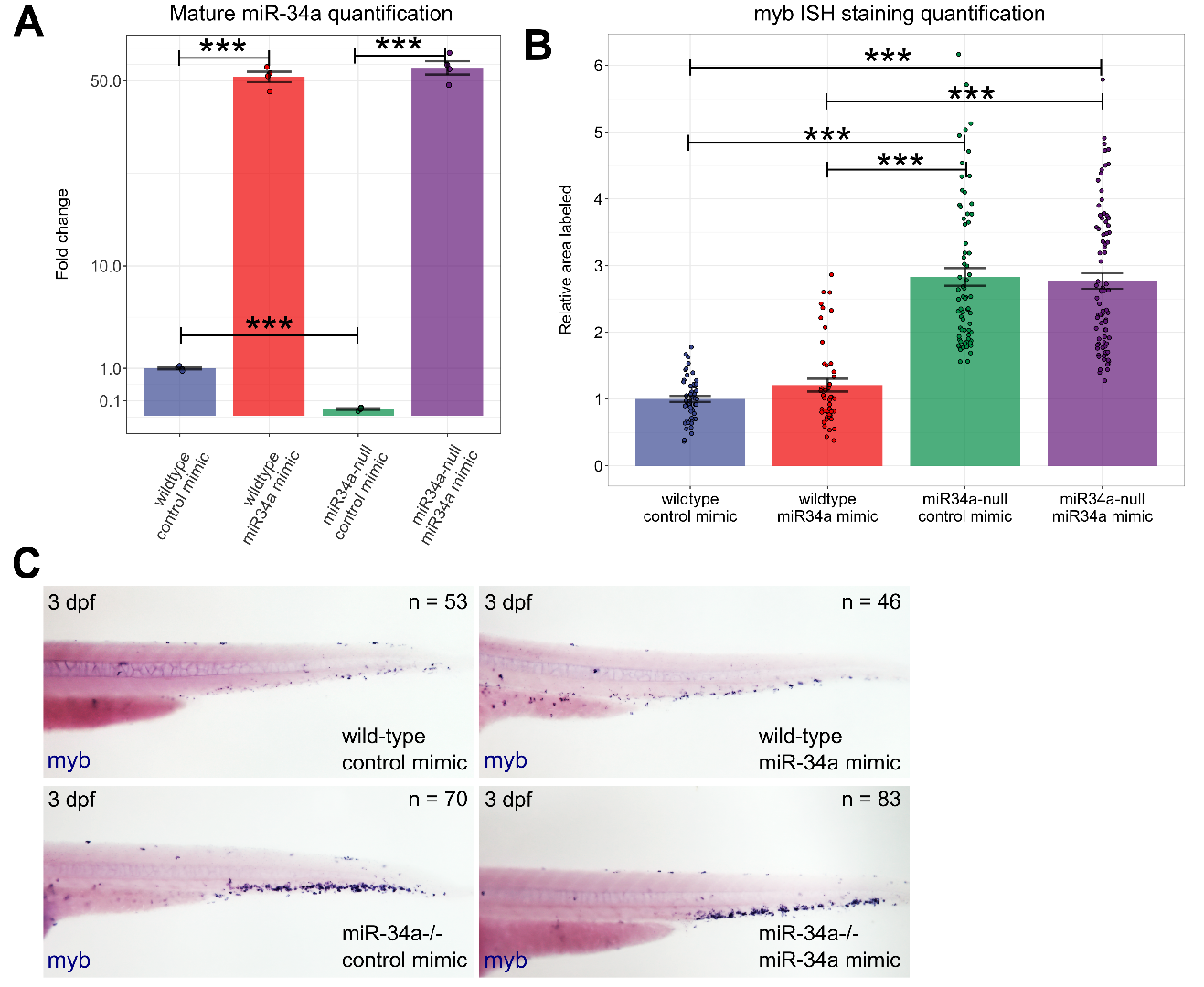
**

**Figure S8. miR-34a mimic does not suppress *myb* expression up-regulation in *miR-34a-/-* mutants.**

(**A**) Mature miR-34a qPCR analysis in 3 dpf wild-type and *miR-34a-/-* embryos injected with control or miR-34a mimics (n = 4 of pooled RNA samples for each condition). (**B**) Quantification of *myb* staining using the Ilastik-Cell Profiler pixel classification approach. Relative areas of positively classified pixels (fold change) are shown. The numbers of embryos are the same as in (**C**). The significances of the differences in (**A**) and (**B**) were determined by one−way ANOVA with a Tukey’s post−hoc test (*** - P-value < 0.001), error bars represent standard errors of the mean, each point represents a pooled RNA sample (A) or an individual stained embryo (**B**). (**C**) Representative *myb* *in situ* staining images of the caudal hematopoietic tissue regions of 3 dpf wild-type and *miR-34a-/-* embryos injected with control or miR-34a mimics. The numbers of embryos are indicated. The staining was performed on embryos from two independent experiments.
