# Supplementary material for: miR-34a is a tumor suppressor in zebrafish and its expression levels impact metabolism, hematopoiesis and DNA damage
Source: PLoS Genet. 2024 May 28;20(5):e1011290. doi: 10.1371/journal.pgen.1011290 (PMC11166285; doi:10.1371/journal.pgen.1011290)
Supplement: S9 Fig — (A) Imaging of TUNEL and DAPI staining of wild-type embryos injected with control or miR-34a mimics and treated with DMSO for 2 hours or 100 nM camptothecin (CPT) for 2 or 3 hours. Developmental stages, treatments and total numbers of imaged and quantified embryos are shown on a representative embryos image. (B) A plot of the relative apoptotic index (TUNEL staining area determined using the Ilastik-Cell Profiler and divided by the embryo-body (without the yolk) DAPI labeling area) for all treatment groups. ANOVA analysis of the injection and treatment factors was done, which is indicated by grouping both CPT-treated samples for both injection types. The significances of the differences between groups were determined by a Tukey’s post−hoc test (**—P-value < 0.01; *—P-value < 0.05), error bars represent standard errors of the mean, each point represents an individual stained embryo. (DOCX) [file pgen.1011290.s011.docx]

**
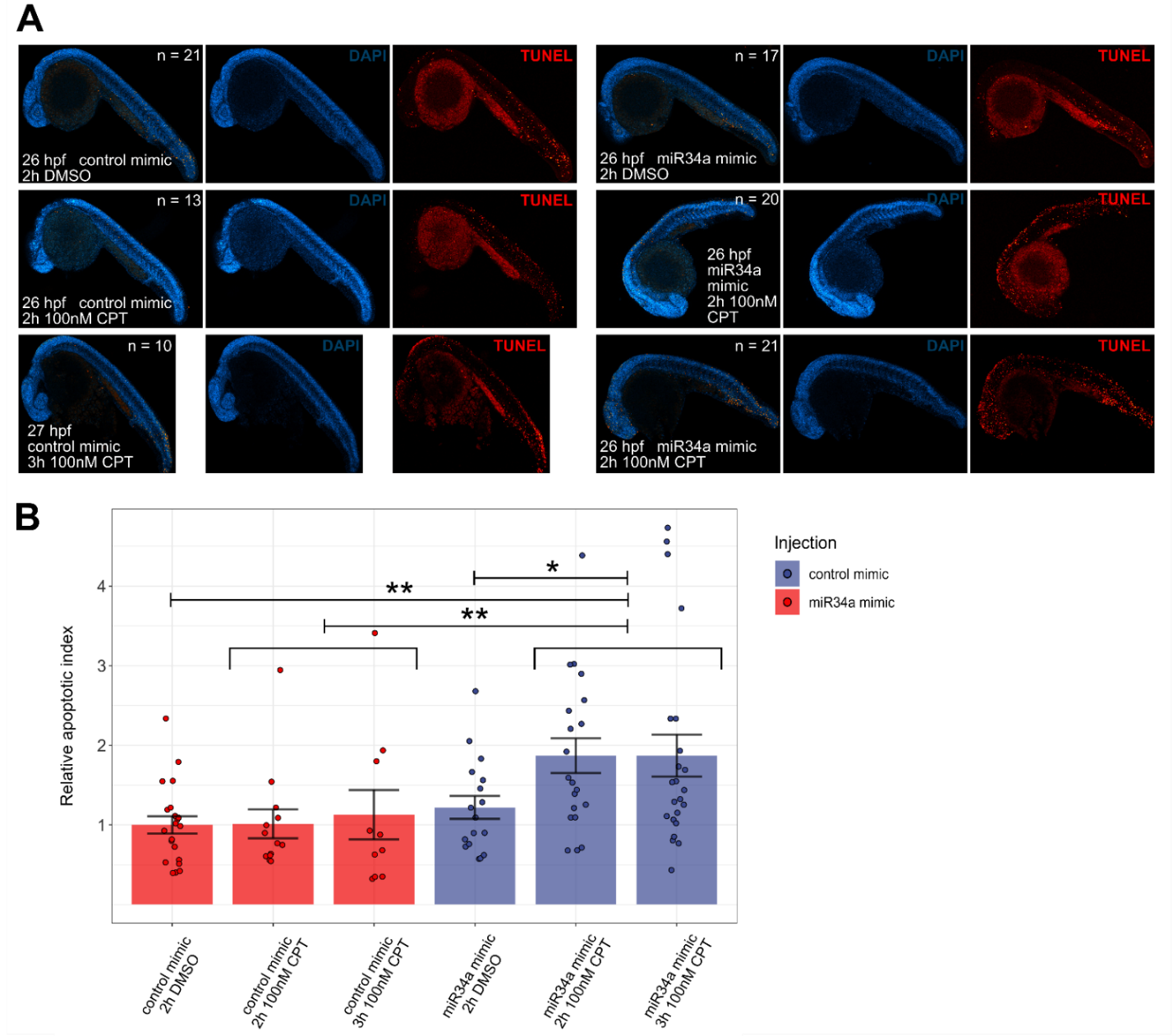
**

**Figure S9. miR-34a over-expression by mimic injection sensitizes zebrafish to earlier apoptosis induction.**

(**A**) Imaging of TUNEL and DAPI staining of wild-type embryos injected with control or miR-34a mimics and treated with DMSO for 2 hours or 100 nM camptothecin (CPT) for 2 or 3 hours. Developmental stages, treatments and total numbers of imaged and quantified embryos are shown on a representative embryos image. (**B**) A plot of the relative apoptotic index (TUNEL staining area determined using the Ilastik-Cell Profiler and divided by the embryo-body (without the yolk) DAPI labeling area) for all treatment groups. ANOVA analysis of the injection and treatment factors was done, which is indicated by grouping both CPT-treated samples for both injection types. The significances of the differences between groups were determined by a Tukey’s post−hoc test (** - P-value < 0.01; * - P-value < 0.05), error bars represent standard errors of the mean, each point represents an individual stained embryo.
