# Supplementary material for: miR-34a is a tumor suppressor in zebrafish and its expression levels impact metabolism, hematopoiesis and DNA damage
Source: PLoS Genet. 2024 May 28;20(5):e1011290. doi: 10.1371/journal.pgen.1011290 (PMC11166285; doi:10.1371/journal.pgen.1011290)
Supplement: S10 Fig — qPCR analysis of 9 genes in 8 hpf wild-type and miR-34a-/- embryos injected with control or miR-34a mimics (n = 6 of pooled RNA samples for each condition). Fold changes relative to the wildtype injected with control mimics are indicated on the y-axis and experimental groups–on the x-axis. ANOVA analysis of the experimental groups vs Cq values was done for each gene. The significances of the differences between groups were determined by a Tukey’s post−hoc test (***—P-value < 0.001; **—P-value < 0.01; *—P-value < 0.05), error bars represent standard errors of the mean, each point represents a pooled RNA sample. Some of the less relevant differences were omitted. The data is from two independent experiments. (DOCX) [file pgen.1011290.s012.docx]

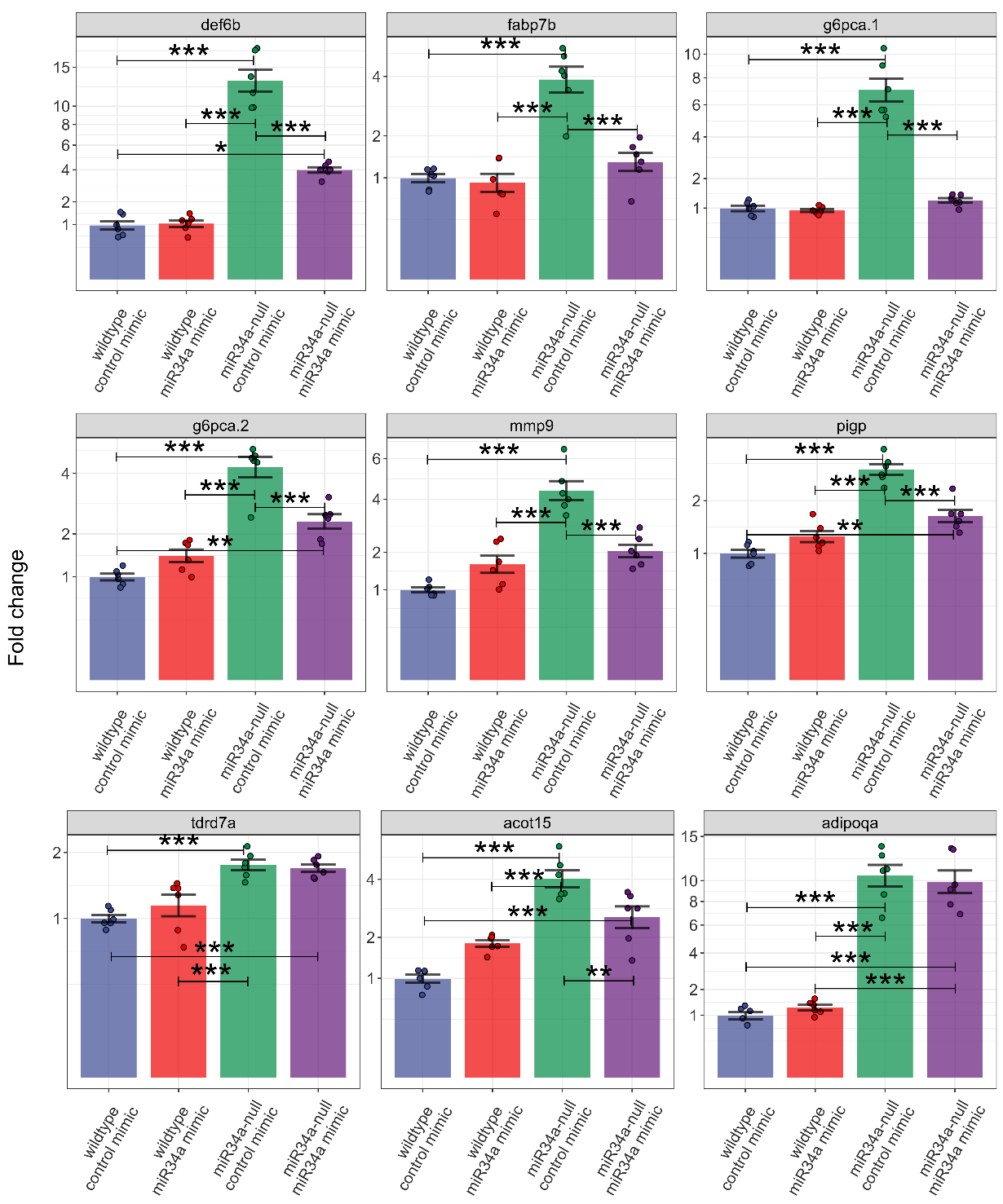


**Figure S10. miR-34a over-expression decreases expression of some genes up-regulated in *miR-34a-/-* mutants at 8 hpf.** qPCR analysis of 9 genes in 8 hpf wild-type and *miR-34a-/-* embryos injected with control or miR-34a mimics (n = 6 of pooled RNA samples for each condition). Fold changes relative to the wildtype injected with control mimics are indicated on the y-axis and experimental groups – on the x-axis. ANOVA analysis of the experimental groups vs Cq values was done for each gene. The significances of the differences between groups were determined by a Tukey’s post−hoc test (*** - P-value < 0.001; ** - P-value < 0.01; * - P-value < 0.05), error bars represent standard errors of the mean, each point represents a pooled RNA sample. Some of the less relevant differences were omitted. The data is from two independent experiments.
