# Supplementary material for: miR-34a is a tumor suppressor in zebrafish and its expression levels impact metabolism, hematopoiesis and DNA damage
Source: PLoS Genet. 2024 May 28;20(5):e1011290. doi: 10.1371/journal.pgen.1011290 (PMC11166285; doi:10.1371/journal.pgen.1011290)
Supplement: S11 Fig — (A) Mature miR-34a (miR34a), p53, p21, puma and mdm2 mRNA levels were analyzed at 28 hpf in control mimic-injected or in miR-34a mimic-injected embryos. The resulting relative expression levels (Fold change) are plotted in the log10 scale. n = 4 for each type of sample. Significance was determined by a t-test and indicated by “***”–P-value < 0.001. (B) and (C) are parts of one experiment repeated twice with 3 biological replicates each aimed at testing if miR-34a over-expression influences p53 target induction by 4-hour treatment with 200 nM camptothecin (CPT). ANOVA was used to measure differences (“***”–P-value < 0.001). (B) Mature miR-34a levels in control mimic-injected or in miR-34a mimic-injected 28 hpf zebrafish embryos treated with 0.005% DMSO or with 200 nM CPT for 4 hours. Levels of mature miR-34a in miR-34a mimic samples are artificial due to injection and “***” indicates their difference from the wild-type samples, but not from each other. (C) p53 target (p53, mdm2, p21, puma, cycG1 and miR34a) expression in control mimic-injected or in miR-34a mimic-injected 28 hpf zebrafish embryos treated with 0.005% DMSO or with 200 nM CPT for 4 hours. Significant induction of the genes is indicated by “***” and signifies the difference in their expression between respective CPT and control samples. None of the other differences were detected. (DOCX) [file pgen.1011290.s013.docx]

**
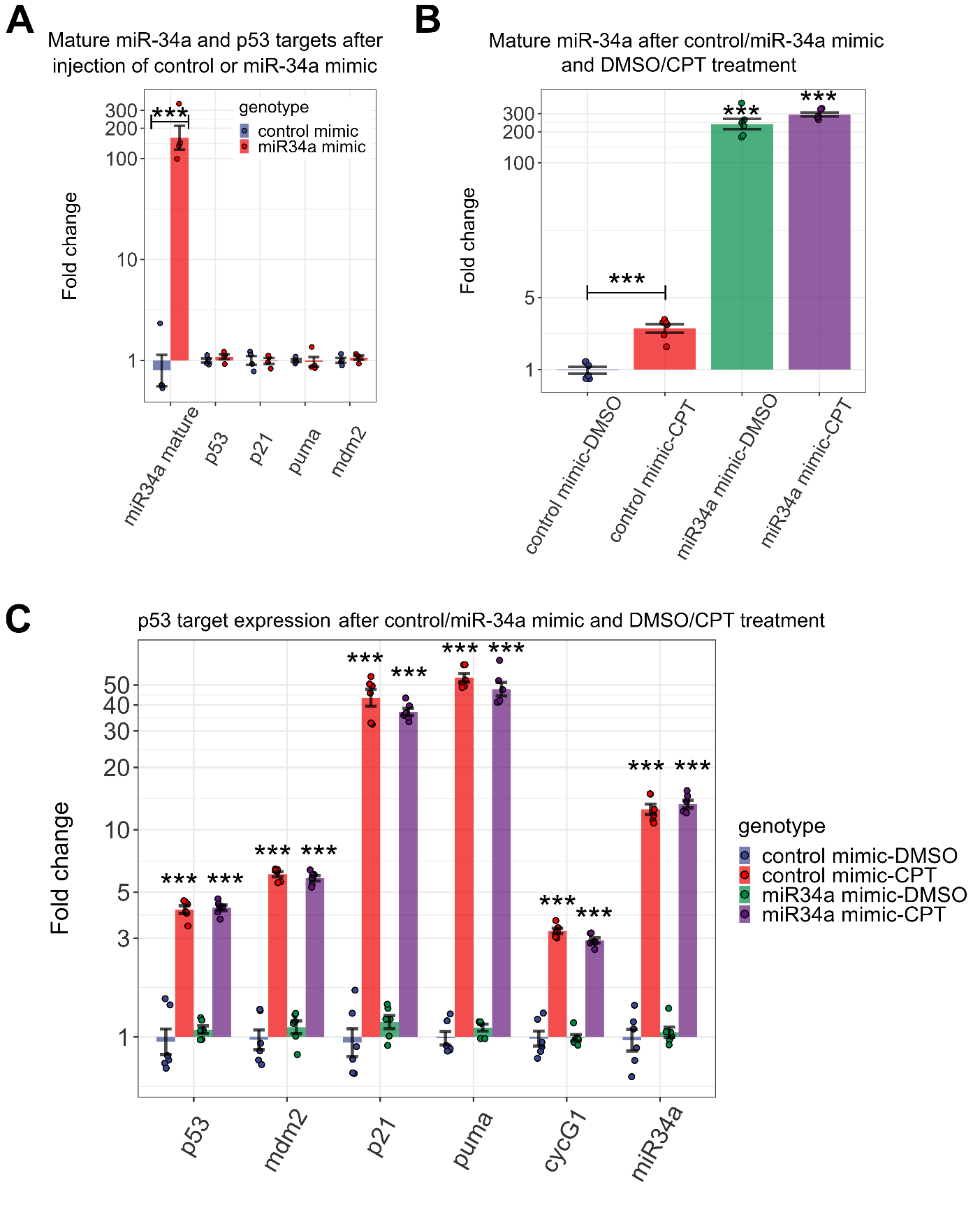
**

**Figure S11. No effect of miR-34a mimic over-expression on p53 target expression at 28 hpf.**

(**A**) Mature miR-34a (miR34a), *p53*, *p21*, *puma* and *mdm2* mRNA levels were analyzed at 28 hpf in control mimic-injected or in miR-34a mimic-injected embryos. The resulting relative expression levels (Fold change) are plotted in the log10 scale. n = 4 for each type of sample. Significance was determined by a t-test and indicated by “***” – P-value < 0.001. (**B**) and (**C**) are parts of one experiment repeated twice with 3 biological replicates each aimed at testing if miR-34a over-expression influences p53 target induction by 4-hour treatment with 200 nM camptothecin (CPT). ANOVA was used to measure differences (“***” – P-value < 0.001). (**B**) Mature miR-34a levels in control mimic-injected or in miR-34a mimic-injected 28 hpf zebrafish embryos treated with 0.005% DMSO or with 200 nM CPT for 4 hours. Levels of mature miR-34a in miR-34a mimic samples are artificial due to injection and “***” indicates their difference from the wild-type samples, but not from each other. (**C**) p53 target (*p53*, *mdm2*, *p21*, *puma*, *cycG1* and *miR34a*) expression in control mimic-injected or in miR-34a mimic-injected 28 hpf zebrafish embryos treated with 0.005% DMSO or with 200 nM CPT for 4 hours. Significant induction of the genes is indicated by “***” and signifies the difference in their expression between respective CPT and control samples. None of the other differences were detected.
